# Random anion distribution in MS_*x*_Se_2−*x*_ (M = Mo, W) crystals and nanosheets†

**DOI:** 10.1039/c8ra01497c

**Published:** 2018-03-09

**Authors:** Minh An T. Nguyen, Arnab Sen Gupta, Jacob Shevrin, Hirofumi Akamatsu, Pengtao Xu, Zhong Lin, Ke Wang, Jun Zhu, Venkatraman Gopalan, Mauricio Terrones, Thomas E. Mallouk

**Affiliations:** Departments of Chemistry, Biochemistry and Molecular Biology, and Physics, The Pennsylvania State University University Park Pennsylvania 16802 USA tem5@psu.edu; Department of Materials Science and Engineering, The Pennsylvania State University University Park Pennsylvania 16802 USA; Department of Physics, The Pennsylvania State University University Park Pennsylvania 16802 USA; Materials Research Institute, The Pennsylvania State University University Park Pennsylvania 16802 USA

## Abstract

The group VIb dichalcogenides (MX_2_, M = Mo, W; X= S, Se) have a layered molybdenite structure in which M atoms are coordinated by a trigonal prism of X atoms. Ternary solid solutions of MS_*x*_Se_2−*x*_ were synthesized, microcrystals were grown by chemical vapor transport, and their morphologies and structures were characterized by using synchrotron X-ray diffraction, Rietveld refinement, DIFFaX simulation of structural disorder, scanning electron microscopy, and energy dispersive X-ray spectroscopy. Double aberration corrected scanning transmission electron microscopy was used to determine the anion distributions in single-layer nanosheets exfoliated from the microcrystals. These experiments indicate that the size difference between S and Se atoms does not result in phase separation, consistent with earlier studies of MX_2_ monolayer sheets grown by chemical vapor deposition. However, stacking faults occur in microcrystals along the layering axis, particularly in sulfur-rich compositions of MS_*x*_Se_2−*x*_ solid solutions.

## Abstract 2

Nanosheets exfoliated from single crystals of the group VIb sulfoselenides (MS*_x_*Se_2−*x*_, M = Mo, W) are solid solutions at the atomic level.

## Graphical abstract

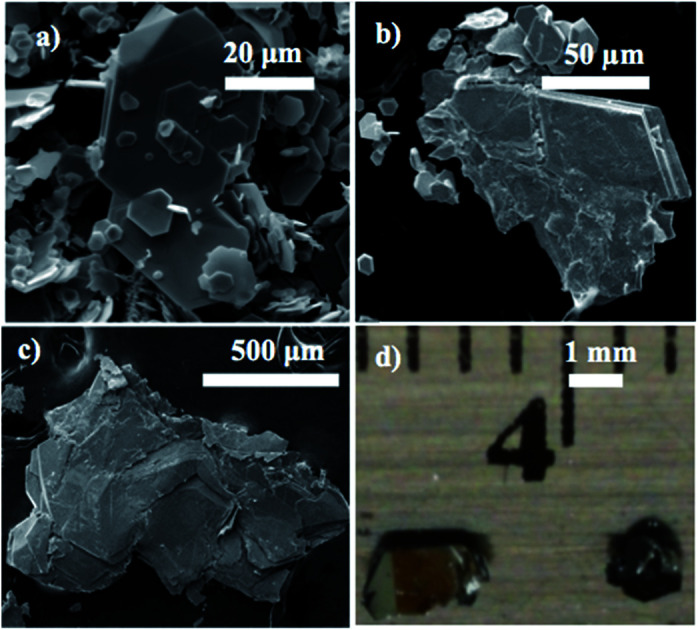

## Introduction

Transition metal dichalcogenides (TMDs) are layered materials with the general formula MX_2_ (M = Mo, W, Nb and X = S, Se, Te). Nanosheets derived from TMDs have been of substantial recent interest in 2-dimensional (2D) materials research. Graphene, the archetypical 2D nanosheet, has very high carrier mobility (>10^5^ cm^2^ V^−1^ s^−1^),^1^ but it has a zero bandgap.^2^ There is a need, especially in electronic devices, for nanosheets that have both non-zero band gaps and high carrier mobility. The physical properties of TMDs cover the range of semiconducting (WS_2_, WSe_2_, MoS_2_, MoSe_2_),^3^ semimetallic (WTe_2_,^4,5^ NbS_2_ (ref. 3)), superconducting (NbS_2_)^6^ and ferromagnetic (Mn intercalated NbS_2_),^7^ and in this way they are complementary to those of graphene. In addition to electronic devices, there is also interest in using TMDs as photoelectrode materials,^8,9^ catalysts,^10–12^ and as platforms for studying charge^13^ and phonon transport.^14–16^

TMDs are also natural candidates as components of multilayer electronic devices made by heterogeneous assembly, such as WS_2_/graphene vertical tunneling transistors,^17^ MoS_2_/WSe_2_ heterojunctions,^18,19^ and alternating MoS_2_/WS_2_ heterostructures.^20^ These devices can have properties and functions that are unique to their composite structure and ultrathin dimensions, resulting, *e.g.*, from correlation of electrons and holes across very thin insulating layers, and from strong spin–orbit coupling that creates an energy gap between spin-up and spin-down valence band states. An enabling feature of TMDs is that they alloy readily with each other because of their similar crystal structures and lattice parameters, thereby providing a route to further tune material properties. The layered hexagonal phases of the MX_2_ (M = Mo, W) disulfides (MS_2_) and diselenides (MSe_2_) are isotypic,^21^ enabling solid solution nanosheets of WS_2_/WSe_2_,^22,23^ MoS_2_/WS_2_,^24,25^ MoS_2_/MoSe_2_,^26^ and WS_2_/MoS_2_ (ref. 27 and 28) alloys to be grown by chemical vapor deposition (CVD). Direct imaging of these monolayers by electron microscopy, as well as photoluminescence spectra, have shown them to be homogeneous at the atomic level,^29–35^ consistent with first-principles calculations.^36,37^ However, a recent study of CVD-grown MS_2_ nanosheets has detected stripes of segregated Mo and W atoms, the formation of which appears to be kinetically controlled.^38^ So far, few such studies have been done with monolayers derived from 3D crystals that are grown under near-equilibrium conditions. Thus, the goal of the present study was to explore the homogeneity and structure of MS_*x*_Se_2−*x*_ nanosheets derived from bulk crystals to determine if nanoscale segregation of phases could be detected.

We present here detailed structural characterization of MS_*x*_Se_2−*x*_ solid solutions by synchrotron X-ray diffraction (SXRD), electron microscopy, and Kelvin probe force microscopy. We find that the bulk materials consist of sheets in which S and Se atoms are randomly distributed over the anion sites, consistent with earlier results from CVD-grown nanosheets. The 3D solids contain stacking faults along the crystallographic *c*-axis, which are most prevalent in sulfur-rich compositions.

## Experimental section

### Solid state synthesis and chemical vapor transport (CVT)

**Fig. 1:**
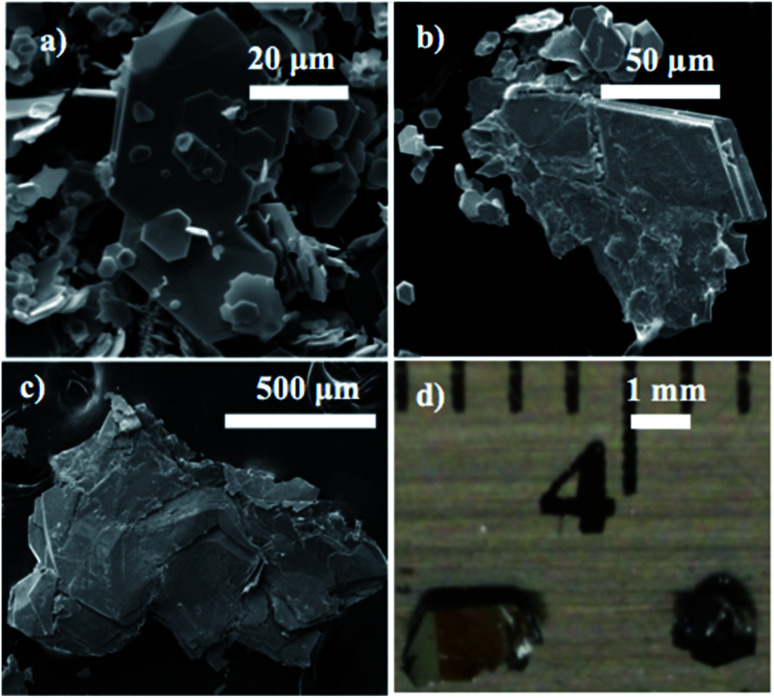
Environmental scanning electron microscope (ESEM) images of (a) WS_0.2_Se_1.8_ powder prior to and (b) following crystal growth by chemical vapor transport (CVT). (c) MoS_0.2_Se_1.8_ crystal grown by CVT. (d) Photograph of typical WS_0.2_Se_1.8_ crystals selected for Kelvin probe force microscopy (KPFM).

The series of MS_*x*_Se_2−*x*_ compounds was synthesized for 0 ≤ *x* ≤ 2 at *x* = 0.2 intervals (10 at%) by mixing stoichiometric amounts of molybdenum or tungsten powder (Acros Organics 99.9%), sublimed sulfur powder (J.T. Baker), and selenium powder (Acros Organics 99.5+%) together and heating at 1000 °C in evacuated and sealed quartz ampoules for 3 days in a tube furnace. Crystallites of the materials thus obtained are polydisperse in size from ≤1 to ≥50 μm. An example is shown in the ESEM micrograph of WS_0.2_Se_1.8_ in Fig. 1.

Solid solution crystals of MS_*x*_Se_2−*x*_ were grown *via* CVT with iodine as the transport agent at ∼1.5 mg cm^−3^ in evacuated and sealed quartz ampoules (10 mm ID, 12 mm OD, 100 mm length) for 10 days. The source and growth zones were kept at 950 °C and 816 °C, respectively for WS_*x*_Se_2−*x*_ and at 950 °C and 870 °C for MoS_*x*_Se_2−*x*_. The resulting crystals were washed with hexane and dried under vacuum to remove solvent and residual iodine. Typical crystals were 100 μm to a few mm in their lateral dimensions as shown in Fig. 1.

### Synchrotron X-ray diffraction, Rietveld analysis and DIFFaX modeling

Ambient temperature synchrotron X-ray diffraction (SXRD) patterns were obtained in the Bragg–Brentano optical system at the 11-BM beamline at Argonne National Laboratory. The calibrated wavelengths of the incident X-rays for the patterns collected were measured to be 0.414179 Å for WS_*x*_Se_2−*x*_ samples with *x* = 0, 1, 1.2, 1.4, 2, 0.413345 Å for samples with *x* = 0.4, 0.6, 0.8, 1.6, and 0.459308 Å for samples with *x* = 0.2 and 1.8. Different wavelengths were used because the diffraction patterns were recorded on different days. For all MoS_*x*_Se_2−*x*_ samples the wavelength was 0.459308 Å. To enable visual comparisons of all patterns for compounds in the series, reciprocal *d*-spacings were plotted instead of 2*θ* values.

Rietveld refinements of the SXRD patterns were done using the RIETAN-FP code.^39^ Refinements were done in the *P*6_3_/*mmc* space group (no. 194) using the modified split pseudo-Voigt function for relaxed reflections mode.^40^ The refinements were first run using the Marquardt method and were refined incrementally to aid in computation speed. Then the conjugate-direction method was used and all variable parameters were refined simultaneously in the final few cycles to ensure that the global minimum had been reached and that the refinement was stable.

The DIFFaX program was utilized to simulate the X-ray lineshapes arising from stacking faults in WS_1.6_Se_0.4_ and MoS_1.6_Se_0.4_.^41^ The ideal atomic positions and unit cell parameters were input from the Rietveld refinement of the SXRD pattern. The planes were translated by (1/3, 2/3) in the *ab* plane in order to introduce stacking faults along the *c*-axis direction. The simulated XRD patterns were calculated using pseudo-Voigt functions for a random distribution of faulted planes.

### Environmental scanning electron microscopy (ESEM) and energy-dispersive X-ray spectroscopy (EDX)

**Fig. 2:**
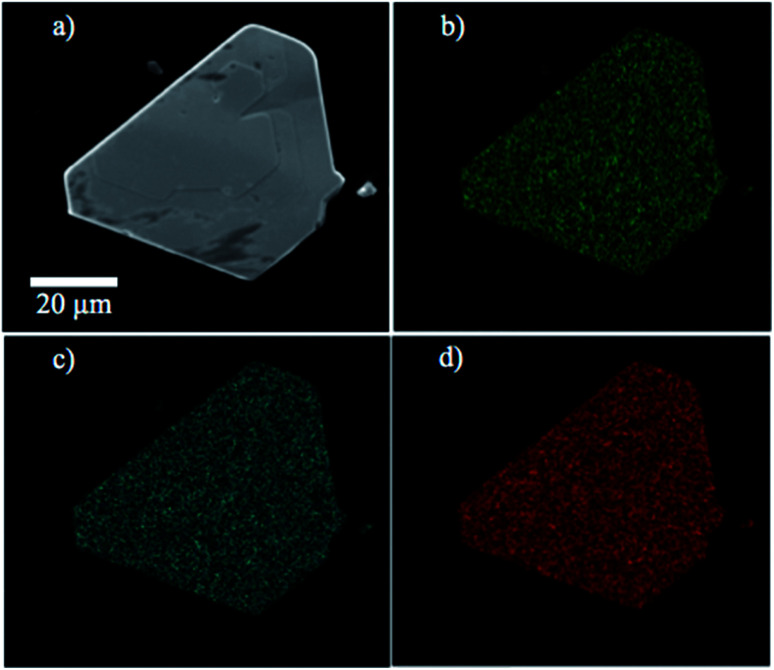
(a) ESEM micrograph of a MoS_0.8_Se_1.2_ crystal and EDX maps of elemental (b) molybdenum, (c) sulfur and (d) selenium content.

ESEM micrographs and EDX experiments were conducted using a FEI Quanta 200 Environmental Scanning Electron Microscope. The samples were deposited onto double-sided carbon tape and mounted onto the sample holder. Representative micrographs of the CVT-grown MoS_*x*_Se_2−*x*_ crystals are shown in Fig. 2 and micrographs of WS_*x*_Se_2−*x*_ crystals are shown in ESI.† Calibrated EDX experiments were performed to analyze the composition and macroscopic homogeneity of the powders made by direct synthesis and crystals grown by CVT.

### Kelvin probe force microscopy (KPFM)

Mono- to few-layer flakes of WS_*x*_Se_2−*x*_ were prepared by micromechanically exfoliating CVT-grown crystals and kish graphite, which was used as a reference, onto the same gold substrate. The gold substrate was first cleaned with ozone and washed thoroughly by sonication in acetone and isopropyl alcohol.

Under an optical microscope, we selected WS_*x*_Se_2−*x*_ and graphite flakes that appeared to be uncontaminated and uniform in thickness for KPFM studies. We further verified the atomic smoothness of the surfaces using tapping mode AFM before performing KPFM measurements.

Standard KPFM techniques were used to measure the contact potential difference Δ*V*_WSSe/Au_ between a WS_*x*_Se_2−*x*_ flake and the gold substrate. Using the same tip, we measured Δ*V*_graphite/Au_ of a graphite flake exfoliated onto the same substrate. The work function of the WS_*x*_Se_2−*x*_ flake is then given by *Φ*_WSSe_ = *Φ*_graphite_ + Δ*V*_WSSe/Au_ − Δ*V*_graphite/Au_. We determined *Φ*_WS_*x*_Se_2−*x*__ using the measured contact potential differences and the known, stable work function of graphite, *Φ*_graphite_ = 4.5 V.^42,43^ This procedure was repeated for many WS_*x*_Se_2−*x*_ flakes exfoliated from bulk crystals of the same nominal chemical composition and of different compositions. Measurements were performed in the ambient on freshly exfoliated flakes. Only flakes with spatially uniform Δ*V* were included in the analysis to minimize the impact of unintentional contamination.

### Scanning transmission electron microscopy and energy-dispersive X-ray spectroscopy

10 mL of a 1 mg mL^−1^ solution of WS_1.8_Se_0.2_ or MoS_1.4_Se_0.6_ powder in 50% ethanol was ultrasonicated with a QSonica Q700 sonicator for 1–2 hours while chilled in an ice/water bath. The resulting suspension was subsequently centrifuged at 6000 rpm for 30 minutes. The supernatant was drop-cast onto a lacy carbon-supported TEM grid. Scanning transmission electron microscopy (STEM) was performed at 80 kV using a FEI Titan^3^ G2 double aberration-corrected microscope. Energy-dispersive X-ray spectroscopic elemental maps of selected monolayers and few-layer regions were also obtained to observe the atomic distribution of tungsten, sulfur, and selenium within the material. The maps were collected by using the superX EDS system on the FEI Titan,^3^ which has four detectors surrounding the sample.

## Results and discussion

### Crystal growth and characterization of WS_*x*_Se_2−*x*_ and MoS_*x*_Se_2−*x*_ solid solutions

Environmental scanning electron microscopy was used to image the morphology of the microcrystalline powders and CVT-grown crystals. A typical example of the size and morphology of the synthesized powder is shown in Fig. 1. Small crystals of the compounds were used for imaging under the ESEM. Typical crystals selected for Kelvin-probe atomic force microscopy measurements of local work functions are shown in Fig. 1d.

Energy-dispersive X-ray spectroscopy experiments were carried out to confirm the composition of the solid solutions. Sulfur, selenium, tungsten, and molybdenum elemental mappings of the solid solutions showed that the elements of interest were homogeneously distributed within the samples within the resolution of the technique, *i.e.*, a few hundred nm. An example of elemental mapping on this length scale is shown in Fig. 2.

### Synchrotron X-ray diffraction, Rietveld analysis and DIFFaX modeling

**Fig. 3:**
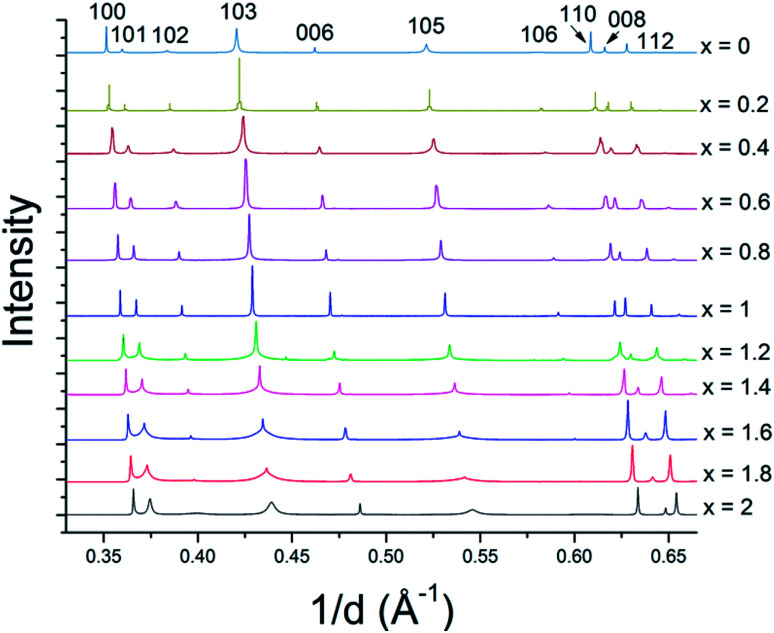
Synchrotron XRD patterns of WS_*x*_Se_2−*x*_ solid solutions. The intensities are normalized to the 100 reflection of WSe_2_. Similar results were obtained with MS_*x*_Se_2−*x*_ solid solutions (see ESI†).

The solid solution powders were characterized by using synchrotron XRD and Rietveld analysis of the resulting diffraction data. The crystal structures of the hexagonal 2H phases of MS_2_ and MSe_2_ (M = Mo, W) are isotypic and the diffraction data are consistent with the formation of solid solutions across the entire composition range. Despite the 17% difference in atomic radii between sulfur and selenium atoms (88 pm and 103 pm, respectively),^44^ the lattice parameters of WS_2_ and WSe_2_ are only slightly mismatched, with *a* constants of 3.1532(4) Å and 3.282(1) Å (a 4% difference) and *c* constants of 12.323(5) Å and 12.96(1) Å (5% difference), respectively.^28^ For MoS_2_ and MoSe_2_ the *a* and *c* lattice parameters differ by only 5% (3.147 *vs.* 3.289 Å, and 12.295 Å *vs.* 12.927 Å).^28^ Based on these differences in atomic radii, the resulting solid solutions are borderline in terms of the Hume-Rothery rules.^35^ The series of bulk MS_*x*_Se_2−*x*_ solid solutions made at *x* = 0.2 intervals (10 at%) are single-phase by X-ray diffraction (see Fig. 3 and ESI†) and could be indexed in space group *P*6_3_/*mmc*. In both the WS_*x*_Se_2−*x*_ and MoS_*x*_Se_2−*x*_ solid solution series, there is an interesting trend in the line shapes in the diffraction patterns, with broadening of the 10*l* reflections for the most sulfur-rich compositions, as shown in Fig. 3.

**Fig. 4:**
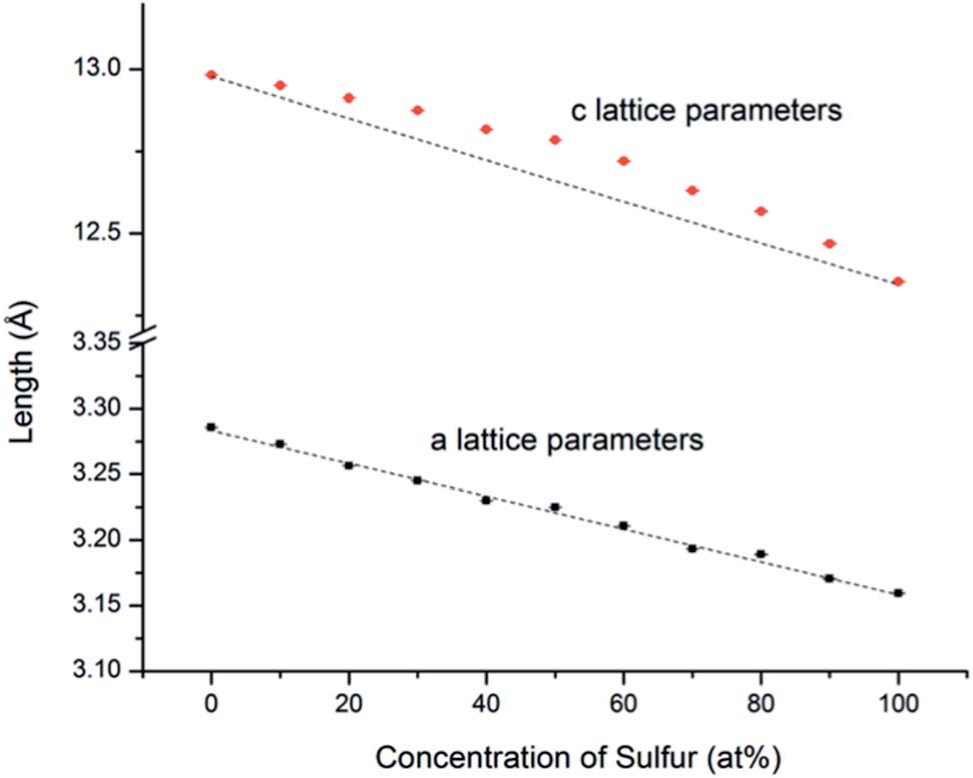
WS_*x*_Se_2−*x*_*a* and *c* lattice parameters determined from Rietveld refinement of SXRD patterns. The black lines represent theoretical values calculated from the end members by using Vegard's law. The error bars are smaller than the size of the symbols. Similar trends were observed with MS_*x*_Se_2−*x*_ solid solutions (see ESI†).

An example of the Rietveld refinement from which lattice parameters were obtained is shown in Fig. S1.1.† From the refined lattice parameters of the end-member compositions, we calculated the lattice parameters (*a* and *c*) that would follow the linear trend of Vegard's law. As shown in Fig. 4 and in ESI,† while the experimental *a* lattice parameters correspond closely to the Vegard's law trend, the experimental *c* lattice parameters show a positive deviation in the middle of the solid solution series; however the *z*-parameter of the chalcogen atoms shows the opposite trend, becoming smallest for compositions near MSSe (M = Mo, W). This compensates the trend in the *c*-axis parameters such that there is little deviation from Vegard's law in terms of the M–X bond lengths.

**Fig. 5:**
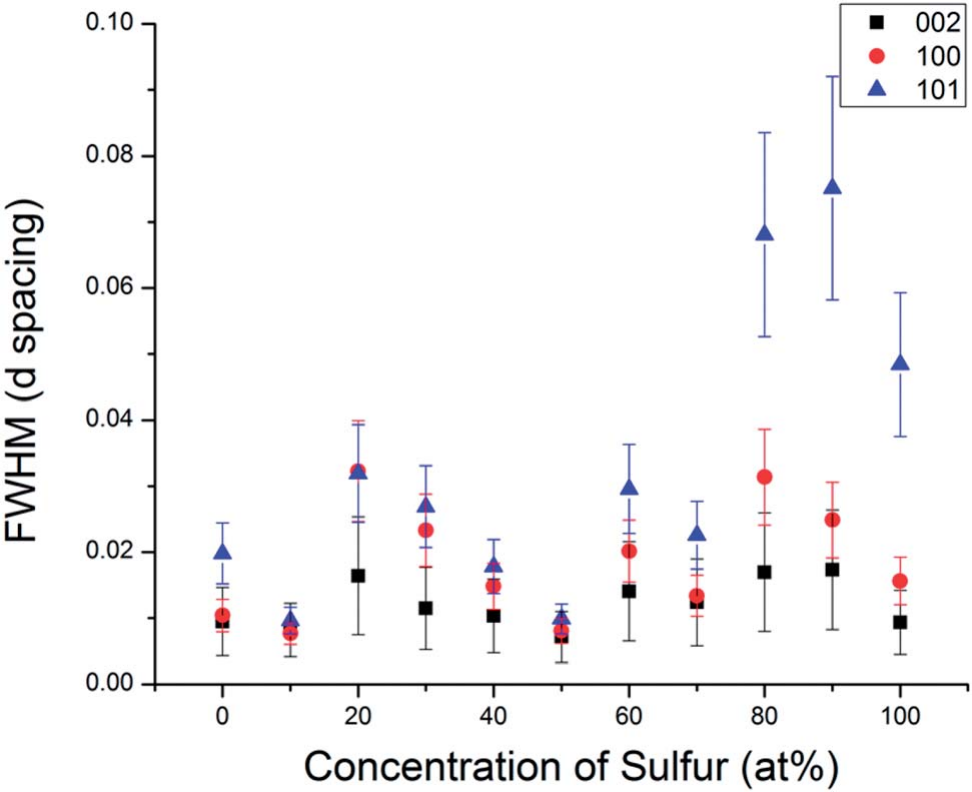
Comparison of the full width at half maximum of different Miller index reflections in the WS_*x*_Se_2−*x*_ series.

In order to understand the nature of structural defects in the solid solution series, we measured the full width at half-maximum (FWHM) of different reflection planes. As shown in Fig. 3 and 5, the 002 reflection (as well as the other 00*l* reflections) do not show a significant broadening effect whereas the *h*0*l* reflections 101, 103, and 104 have non-Gaussian peak shapes, especially for sulfur-rich solid solutions. The effect is quite pronounced for the end-member compositions WS_2_ and MoS_2_. In addition, the 100 reflection shows signs of broadening when the solid solutions contain predominantly either sulfur or selenium.

From Fig. 3 and 5, it is evident that reflections with *h* − *k* = 3*N* ± 1 are broadened, where *N* is an integer. Following the analysis of Warren,^46^ in the hexagonal system *hkl* reflections that have *h* − *k* = 3 *N* are not broadened by along the *c*-axis, whereas those with *h* − *k* = 3*N* ± 1 are. It is also worth noting that no displacement or peak asymmetry is expected from these kinds of stacking faults. The symmetric shape of the observed *h*0*l* reflections supports the conclusion that the broadening arises primarily from stacking faults rather than from strain or particle size effects. The experimental and theoretical results of Delmas and Tessier on growth and deformation of stacking faults in layered Ni(OH)_2_ show similar line shapes to those that we observe in the MS_*x*_Se_2−*x*_ solid solutions at high concentration of sulfur, suggesting a similar structural origin.^47^

**Fig. 6:**
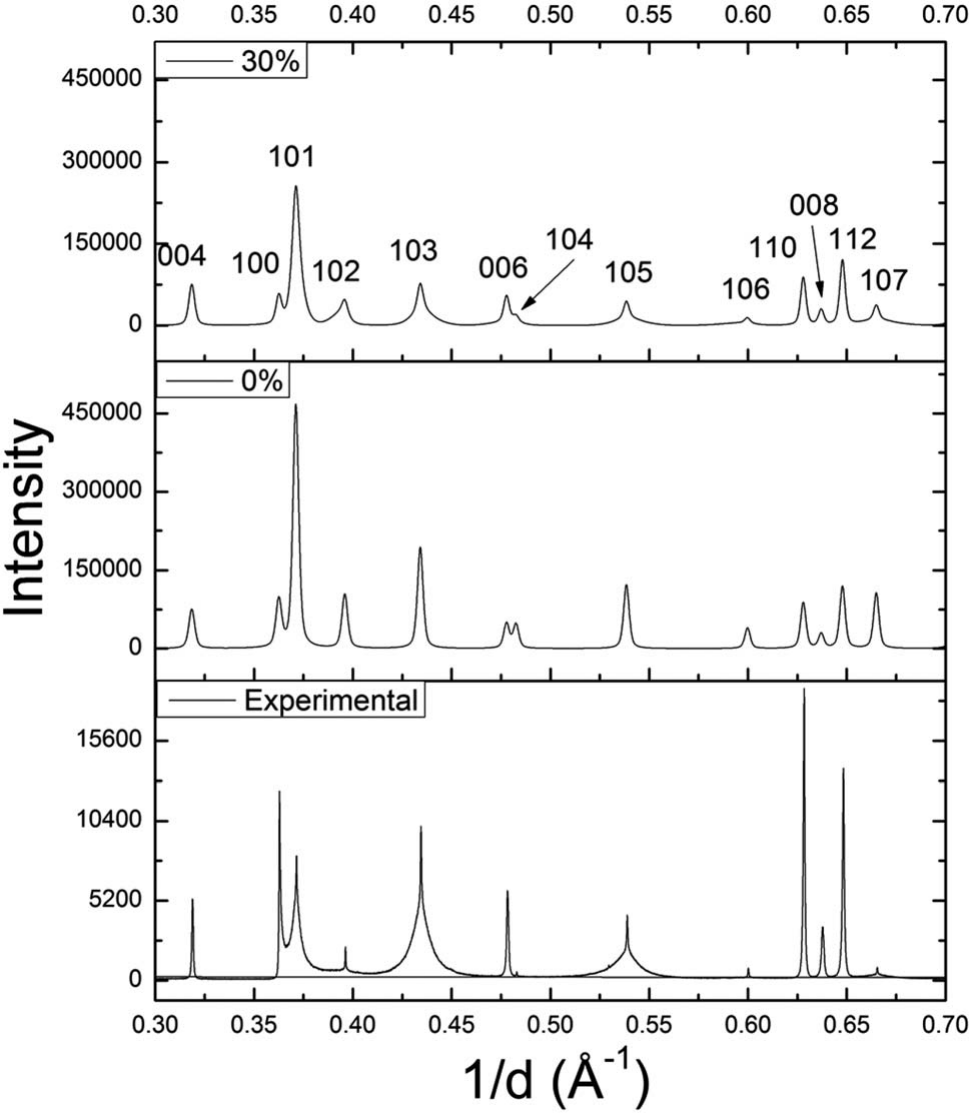
DIFFaX simulated XRD patterns of WS_1.6_Se_0.4_ with 0% and 30% stacking faults, and the experimental SXRD pattern.

Using DIFFaX, stacking faults along the *c*-axis were modeled for the WS_1.6_Se_0.4_ and MoS_1.6_Se_0.4_ compositions, which have particularly broad 10*l* lines. The simulated pattern with 30% stacking faults (Fig. 6) showed qualitatively similar line shapes to the experimental SXRD pattern. The relative intensities and widths of the 00*l* reflections were largely unaffected whereas the 10*l* reflections became progressively broader as the density of stacking faults was increased in the simulation. Similar results were obtained for MoS_1.6_Se_0.4_ (see ESI†).

### Kelvin probe force microscopy

**Fig. 7:**
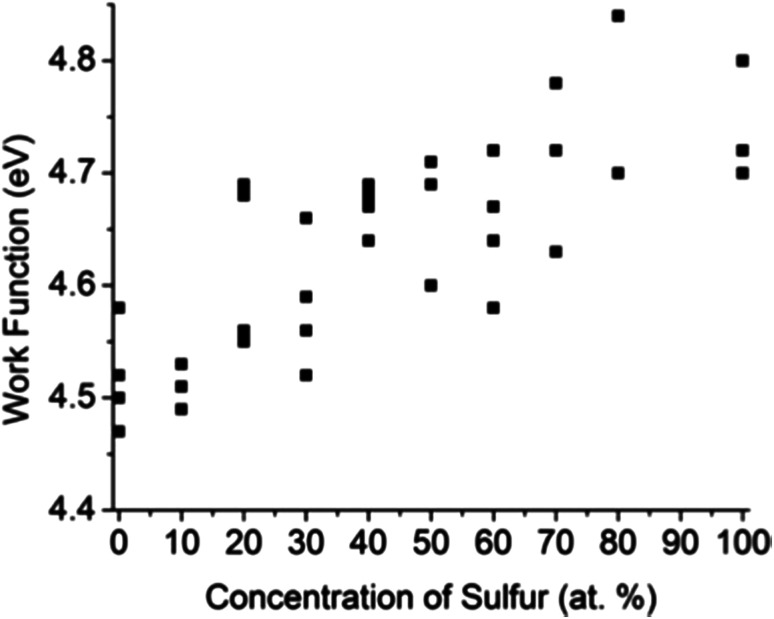
Work functions of mechanically exfoliated WS_*x*_Se_2−*x*_ flakes measured by KPFM. The typical error in individual measurements was 0.01–0.02 eV.

Kelvin probe force microscopy (KPFM) was utilized to measure the work function *Φ* local to small flakes of the solid solution materials. The results for WS_*x*_Se_2−*x*_ solid solutions are plotted in Fig. 7. Each data point corresponds to a different flake. As the value of *x* increased from *x* = 0 to *x* = 2, the overall trend of *Φ* shows a smooth increase, in good agreement with previous theoretical calculations.^48^ The data also show variations of *Φ* at same bulk sulfur concentration, the magnitude of which is larger than a range of 0.06 V observed in the variation of the Au/graphite contact potential difference. This observation suggests that the work function of WS_*x*_Se_2−*x*_ is sensitive to the local surface chemistry of individual flakes.

### Scanning transmission electron microscopy and energy-dispersive X-ray spectroscopy

**Fig. 8:**
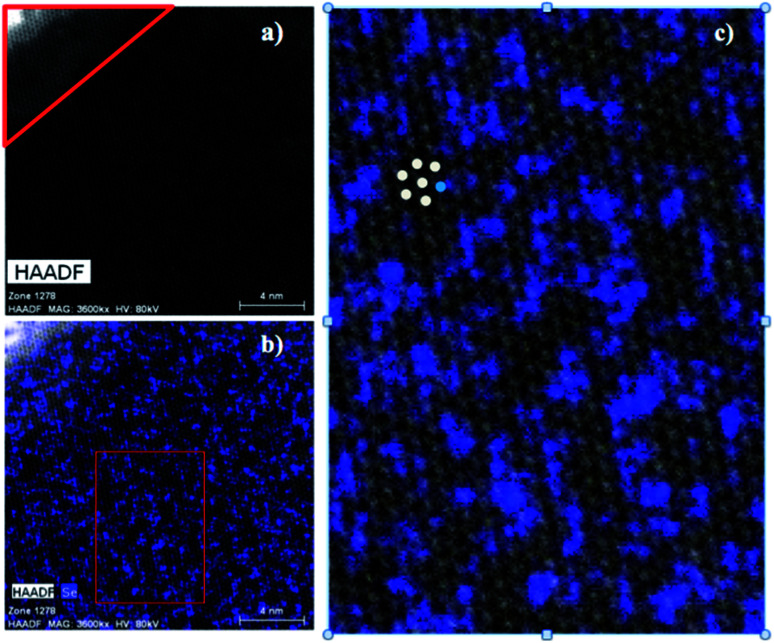
(a) HAADF-STEM micrograph of WS_1.8_Se_0.2_. The corner outlined in red is a multilayer region. The rest of the image is a monolayer region. Elemental EDX maps of (b) selenium overlayed on the HAADF-STEM micrograph of WS_1.8_Se_0.2_. The red box region in (b) was used for quantifying the degree of clustering within the monolayer. (c) The enlarged red box region shown from (b) with colored dots added to illustrate the nearest-neighbor counting process.

In order to probe the possibility of nanoscale phase separation in nanosheets derived from the microcrystals, monolayer regions of individual flakes were located under the TEM using high angle annular dark field scanning transmission electron microscopy (HAADF-STEM). A representative set of images is shown in Fig. 8. We chose the WS_1.8_Se_0.2_ and MoS_1.8_Se_0.2_ compositions to image because they had relatively broad lines in the bulk XRD patterns, and thus were most likely to show phase segregation. An elemental EDX map of selenium in WS_1.8_Se_0.2_ (Fig. 8b and c) shows the distribution of Se atoms, which are projected from both sides of the monolayer sheet. Although the individual Se atoms are not resolved, the overlay of the HAADF-STEM and EDX maps enables us to identify the metal atoms (light gray) that are coordinated to no Se atoms, and therefore to six S atoms. These atoms can be differentiated from metal atoms that are coordinated to at least one Se atom, although we cannot quantitatively distinguish those coordinated to 1, 2, 3, 4, 5, and 6 Se atoms.

To quantify the possible clustering of MS_2_ and MSe_2_ phases within the monolayer, we applied two statistical methods. First, we counted the metal atoms coordinated to at least one Se atom and compared that number to those that are coordinated to S atoms only. Statistically, if the Se atoms are randomly distributed, then at a composition of MS_1.8_Se_0.2_ the probability that any M atom is coordinated to six S atoms is (0.90)^6^ = 0.53. Experimentally we find that the light gray atoms comprise 51% of the atoms in the WS_1.8_Se_0.2_ image, consistent with a random distribution. A similar analysis for MoS_1.8_Se_0.2_ (see ESI†) also gave 51%. Second, we applied the nearest-neighbor counting method developed by Hwang *et al.* for alloy nanoparticles^49^ and later used by Dumcenco *et al.* to analyze clustering in Mo_1−*x*_W_*x*_S_2_ nanosheets.^28^ For this analysis, the numbers of light gray and blue nearest neighbors of each metal atom in the overlay image (Fig. 8c) were counted. For example, in Fig. 8c the overlaid colored dots illustrate a light gray W atom that is surrounded by five other light gray W atoms (which are coordinated to S only) and one blue W atom (which is coordinated to one or more Se atoms). The clustering of light gray W atoms can be quantified by a parameter *J*, where *J* = 0% corresponds to complete separation into WS_2_ and WSe_2_ microphases. *J* = 100% is expected for a random distribution of blue and light gray W atoms, and higher *J* values imply avoidance of gray–gray and blue–blue nearest neighbor pairs:^49^
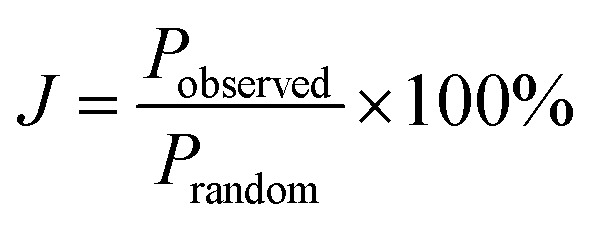
here *P*_observed_ is defined as the ratio of the average number of blue nearest neighbors to the total number of nearest neighbors (six), and *P*_random_ is the fraction of blue atoms in the image (see ESI† for details).

The *J* value calculated from 641 tungsten atoms in the overlay image (Fig. 8c) is 84 ± 4%. The same analysis for 549 molybdenum atoms in MoS_1.8_Se_0.2_ gave *J* = 70 ± 5%. These values are close to the value of 100% expected for a random distribution of Se in the monolayer, and are consistent with the simpler calculation based on the stoichiometry and the ratio of light gray- to blue-colored M atoms in the overlay image.

### Photoluminescence spectroscopy

**Fig. 9:**
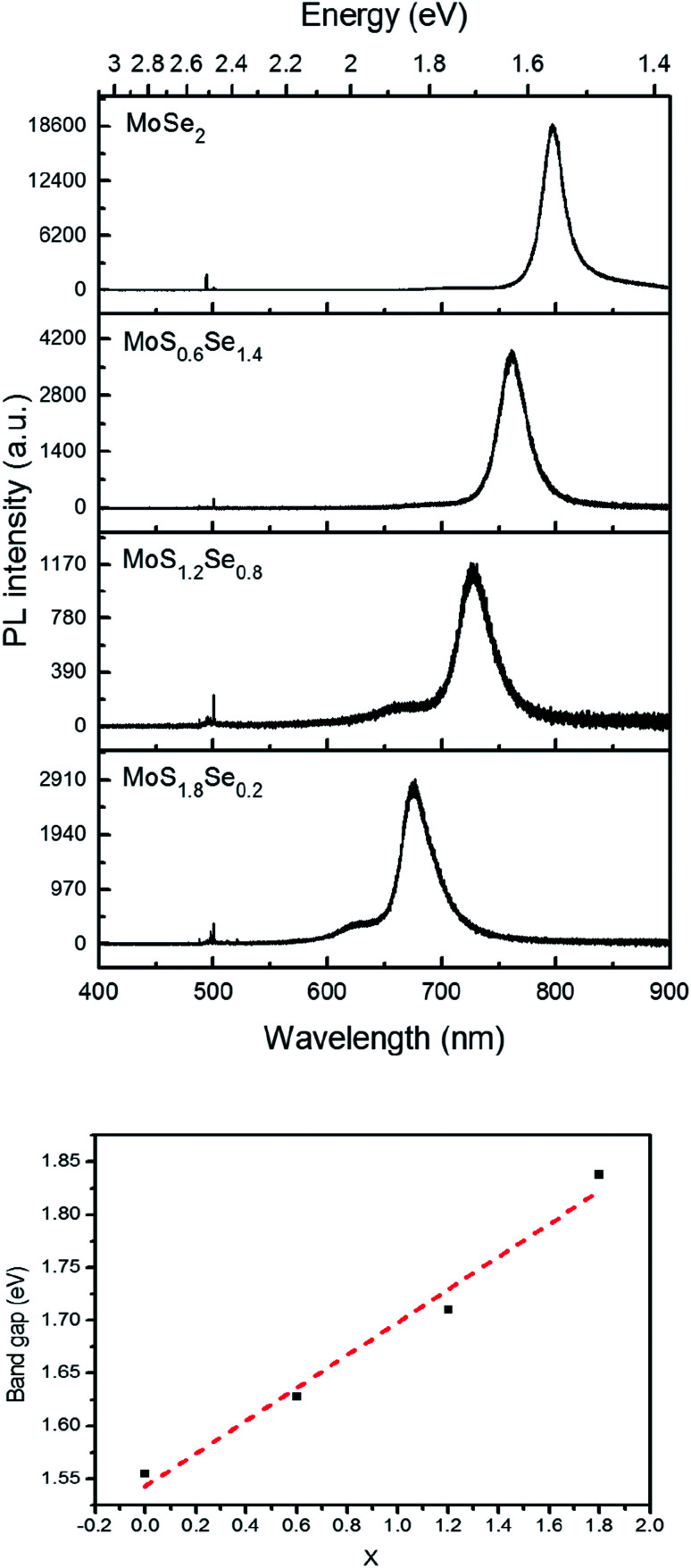
Top: Photoluminescence spectra of MoS_*x*_Se_2−*x*_ monolayers with 488 nm laser excitation. Bottom: Vegard's law plot of optical bandgap *vs. x* in MoS_*x*_Se_2−*x*_.

Monolayer samples of MoS_*x*_Se_2−*x*_ were also interrogated by photoluminescence spectroscopy in order to determine if S and Se atoms were homogeneously distributed in the sheet. Only one dominant peak was observed in the photoluminescence spectra, again consistent with the idea that there is no separation into S- and Se-rich domains on the micron length scale of the optical probe (Fig. 9). As the Se concentration increases, the photoluminescence peak position red-shifts, from 677 nm (1.83 eV) for MoS_1.8_Se_0.2_ to 726 nm (1.71 eV), 761 nm (1.63 eV), and 797 nm (1.56 eV) for MoS_1.6_Se_0.4_, MoS_1.2_Se_0.8_, MoS_0.6_Se_1.2_, and MoS_0.2_Se_1.8_ respectively. The optical band gap derived from these spectra is plotted as a function of sulfur concentration in Fig. 9. The linear trend follows Vegard's law as expected for homogeneous samples.

## Conclusions

Solid solutions of WS_*x*_Se_2−*x*_ and MoS_*x*_Se_2−*x*_ with 0 ≤ *x* ≤ 2 were synthesized at *x* = 0.2 intervals (10 at%) as powders and grown as crystals by CVT. The solid solutions have homogeneous compositions on the micrometer length scale, as determined by SXRD, SEM and SEM-EDS. Stacking faults in sulfur-rich members of the solid solution series are indicated by line widths and line shapes in XRD patterns and confirmed by DIFFaX modeling of the diffraction data. HAADF-STEM and KPFM measurements further demonstrate that the bulk materials and individual nanosheets derived from them are homogeneous solid solutions on the nanometer length scale. Because CVT is a near-equilibrium growth method, we can conclude that the homogeneous solid solution is thermodynamically stable relative to a micro-phase separated material, despite the lattice strain induced by the difference in the atomic radii of S and Se.

## Conflicts of interest

There are no conflicts to declare.

## Supplementary Material

RA-008-C8RA01497C-s001
