# Supplementary material for: Random anion distribution in MSxSe2−x (M = Mo, W) crystals and nanosheets
Source: RSC Adv. 2018 Mar 9;8(18):9871–8. doi: 10.1039/c8ra01497c (PMC9078701; doi:10.1039/c8ra01497c)
Supplement: RA-008-C8RA01497C-s001 [file RA-008-C8RA01497C-s001.pdf]

Electronic Supplementary Material (ESI) for RSC Advances.
This journal is © The Royal Society of Chemistry 2018

# Random Anion Distribution in $MS_xSe_{2-x}$ (M=Mo, W) Crystals and Nanosheets

Minh An T. Nguyen,[a] Arnab Sen Gupta,[b] Jacob Shevrin,[c] Hirofumi Akamatsu,[b] Pengtao Xu,[a] Zhong Lin,[c] Ke Wang,[d] Jun Zhu,[c] Venkatraman Gopalan,[b] Mauricio Terrones[c] and Thomas E. Mallouk*[a,c]

[a]Departments of Chemistry, Biochemistry and Molecular Biology, and Physics, The Pennsylvania State University, University Park, Pennsylvania 16802, USA.
[b]Department of Materials Science and Engineering, The Pennsylvania State University, University Park, Pennsylvania 16802, USA.
[c]Department of Physics, The Pennsylvania State University, University Park, Pennsylvania 16802, USA.
* Corresponding author, email tem5@psu.edu.

**SUPPORTING INFORMATION**

**Table of contents**

1. Rietveld refinement of synchrotron X-ray powder diffraction data S-1

2. Characterization of crystal morphology and composition S-3

3. HAADF-EDX mapping of atomic distributions of W, S, and Se S-10

4. Calculation of the clustering parameter J from HAADF-EDX data S-12

## 1. Rietveld refinement of synchrotron X-ray powder diffraction data

Rietveld refinements were carried out as described in the text. The z-parameters for S and Se were allowed to float throughout the refinement. However, they were kept equal to each other to maintain $P6_3/mmc$ symmetry. Preferred orientation along the c-axis was used as a parameter in the refinement. A preferred orientation of 1 is for an ideal case where there is no preferred orientation. Examples of refined XRD patterns are shown in Figures S1 and S2.

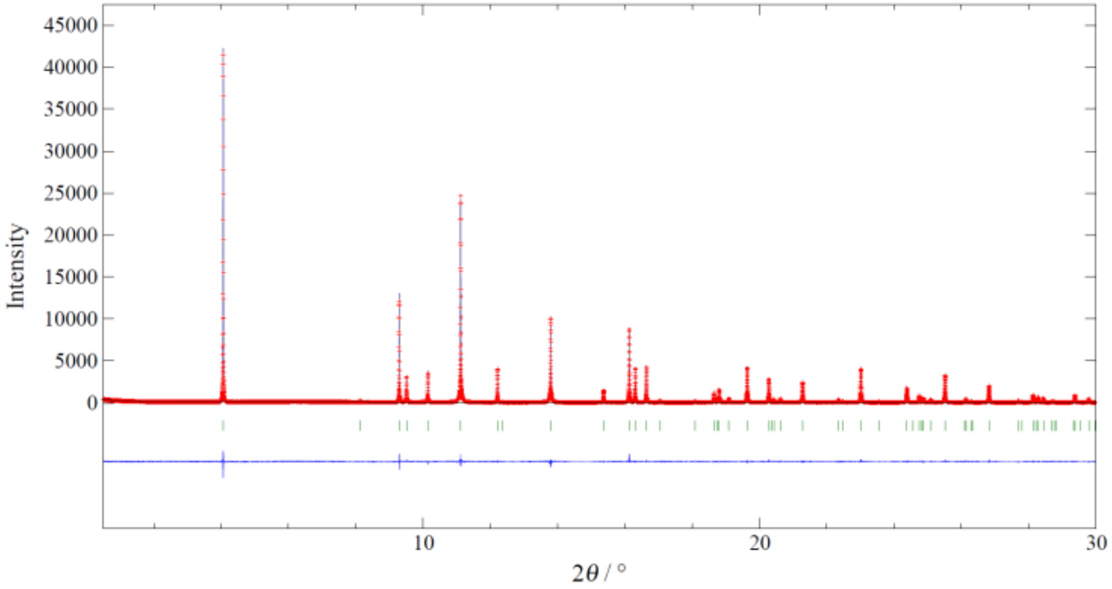


Figure S1.1. Rietveld refinement of $WS_{0.2}Se_{1.8}$.

Table S1.1. Rietveld refinement results for $WS_xSe_{2-x}$ solid solutions.

| Sample | Atom | Site | x | y | z | Preferred orientation |
|---|---|---|---|---|---|---|
| $WS_2$ | W1 | 2d | 2/3 | 1/3 | 0.25 | 0.999853 |
| a/b = 3.15939 ± 0.00003 | S1 | 4f | 1/3 | 2/3 | 0.122843 | |
| c = 12.3519 ± 0.00015 | | | | | | |
| $R_p$ = 7.9 | | | | | | |
| $WS_{1.8}Se_{0.2}$ | W1 | 2d | 2/3 | 1/3 | 0.25 | 0.900451 |
| a/b = 3.17018 ± 0.00004 | S1/S | 4f | 1/3 | 2/3 | 0.122554 | |
| c = 12.46440 ± 0.00027 | S2/Se | 4f | 1/3 | 1/3 | 0.122554 | |
| $R_p$ = 7.1 | | | | | | |
| $WS_{1.6}Se_{0.4}$ | W1 | 2d | 2/3 | 1/3 | 0.25 | 1.00417 |

| | | | | | | |
|---|---|---|---|---|---|---|
| a/b = 3.18468 ± 0.00007 | S1/S | 4f | 1/3 | 2/3 | 0.120308 | |
| c = 12.47804 ± 0.0004 | S2/Se | 4f | 1/3 | 2/3 | 0.120308 | |
| $R_p$ = 8.1 | | | | | | |
| $WS_{1.4}Se_{0.6}$ | W1 | 2d | 2/3 | 1/3 | 0.25 | 1.05556 |
| a/b = 3.19733 ± 0.00004 | S1/S | 4f | 1/3 | 2/3 | 0.118441 | |
| c = 12.54111 ± 0.0002 | S2/Se | 4f | 1/3 | 2/3 | 0.118441 | |
| $R_p$ = 8.6 | | | | | | |
| $WS_{1.2}Se_{0.8}$ | W1 | 2d | 2/3 | 1/3 | 0.25 | 1.08693 |
| a/b = 3.20997 ± 0.00005 | S1/S | 4f | 1/3 | 2/3 | 0.119787 | |
| c = 12.60418 ± 0.00026 | S2/Se | 4f | 1/3 | 2/3 | 0.119787 | |
| $R_p$ = 9.9 | | | | | | |
| WSSe | W1 | 2d | 2/3 | 1/3 | 0.25 | 1.7259 |
| a/b = 3.22261 ± 0.00002 | S1/S | 4f | 1/3 | 2/3 | 0.11674 | |
| c = 12.66725 ± 0.0001 | S2/Se | 4f | 1/3 | 2/3 | 0.11674 | |
| $R_p$ = 11.9 | | | | | | |
| $WS_{0.8}Se_{1.2}$ | W1 | 2d | 2/3 | 1/3 | 0.25 | |
| a/b = 3.23526 ± 0.00004 | S1/S | 4f | 1/3 | 2/3 | 0.119199 | |
| c = 12.73032 ± 0.00015 | S2/Se | 4f | 1/3 | 2/3 | 0.119199 | |
| $R_p$ = 10.0 | | | | | | |
| $WS_{0.6}Se_{1.4}$ | W1 | 2d | 2/3 | 1/3 | 0.25 | 1.04354 |
| a/b = 3.24790 ± 0.00004 | S1/S | 4f | 1/3 | 2/3 | 0.120658 | |
| c = 12.79339 ± 0.00015 | S2/Se | 4f | 1/3 | 2/3 | 0.120658 | |
| $R_p$ = 9.4 | | | | | | |
| $WS_{0.4}Se_{1.6}$ | W1 | 2d | 2/3 | 1/3 | 0.25 | 1.33194 |
| a/b = 3.26055 ± 0.00004 | S1/S | 4f | 1/3 | 2/3 | 0.120825 | |
| c = 12.85646 ± 0.00024 | S2/Se | 4f | 1/3 | 2/3 | 0.120825 | |
| $R_p$ = 8.0 | | | | | | |
| $WS_{0.2}Se_{1.8}$ | W1 | 2d | 2/3 | 1/3 | 0.25 | 0.980661 |
| a/b = 3.27319 ± 0.00002 | S1/S | 4f | 1/3 | 2/3 | 0.120299 | |
| c = 12.91953 ± 0.00005 | S2/Se | 4f | 1/3 | 2/3 | 0.120299 | |
| $R_p$ = 8.4 | | | | | | |
| $WSe_2$ | W1 | 2d | 2/3 | 1/3 | 0.25 | 0.995351 |
| a/b = 3.28584 ± 0.00001 | S1/S | 4f | 1/3 | 2/3 | 0.121394 | |
| c = 12.98260 ± 0.00008 | S2/Se | 4f | 1/3 | 2/3 | 0.121394 | |
| $R_p$ = 8.0 | | | | | | |

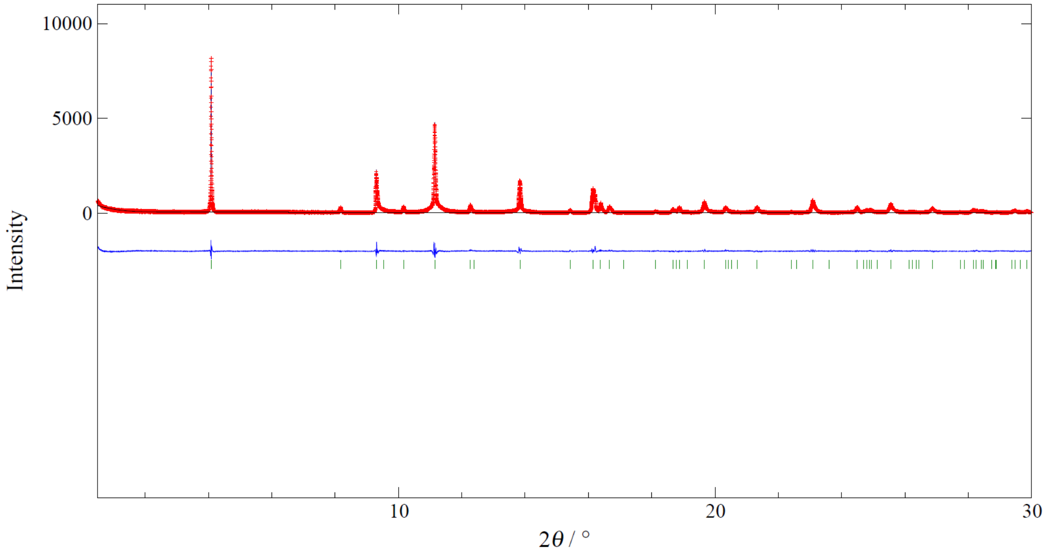


Figure S1.2. Rietveld refinement of $MoS_{0.2}Se_{1.8}$.

Table S1.2. Rietveld refinement results for $MoS_xSe_{2-x}$ solid solutions.

| Sample | Atom | Site | x | y | z | Preferred orientation |
|---|---|---|---|---|---|---|
| $MoS_2$ | Mo1 | 2c | 1/3 | 2/3 | 0.25 | 1.01537 |
| a/b = 3.16224 ± 0.00011 | S1 | 4f | 1/3 | 2/3 | 0.624611 | |
| c = 12.29399 ± 0.00090 | | | | | | |
| $R_p$ = 5.37 | | | | | | |
| $MoS_{1.8}Se_{0.2}$ | Mo1 | 2c | 1/3 | 2/3 | 0.25 | 0.909656 |
| a/b = 3.17291 ± 0.00007 | S1/S | 4f | 1/3 | 2/3 | 0.622098 | |
| c = 12.37002 ± 0.00045 | S2/Se | 4f | 1/3 | 2/3 | 0.622098 | |
| $R_p$ = 12.4 | | | | | | |
| $MoS_{1.6}Se_{0.4}$ | Mo1 | 2c | 1/3 | 2/3 | 0.25 | 0.863259 |
| a/b = 3.18372 ± 0.00004 | Mo1/S | 4f | 1/3 | 2/3 | 0.620147 | |
| c = 12.47169 ± 0.00020 | S2/Se | 4f | 1/3 | 2/3 | 0.620147 | |
| $R_p$ = 12.2 | | | | | | |
| $MoS_{1.4}Se_{0.6}$ | Mo1 | 2c | 1/3 | 2/3 | 0.25 | 0.913242 |
| a/b = 3.19442 ± 0.00003 | S1/S | 4f | 1/3 | 2/3 | 0.619961 | |
| c = 12.54590 ± 0.00017 | S2/Se | 4f | 1/3 | 2/3 | 0.619961 | |
| $R_p$ = 10.6 | | | | | | |
| $MoS_{1.2}Se_{0.8}$ | Mo1 | 2c | 1/3 | 2/3 | 0.25 | 0.913567 |

| | | | | | | |
|---|---|---|---|---|---|---|
| a/b = 3.20475 ± 0.00004 | S1/S | 4f | 1/3 | 2/3 | 0.620549 | |
| c = 12.61090 ± 0.00021 | S2/Se | 4f | 1/3 | 2/3 | 0.620549 | |
| $R_p$ = 9.32 | | | | | | |
| MoSSe | Mo1 | 2c | 1/3 | 2/3 | 0.25 | 1.56051 |
| a/b = 3.22052 ± 0.00006 | S1/S | 4f | 1/3 | 2/3 | 0.61985 | |
| c = 12.69650 ± 0.00023 | S2/Se | 4f | 1/3 | 2/3 | 0.61985 | |
| $R_p$ = 11.6 | | | | | | |
| $MoS_{0.8}Se_{1.2}$ | Mo1 | 2c | 1/3 | 2/3 | 0.25 | 1.31168 |
| a/b = 3.23190 ± 0.00006 | S1/S | 4f | 1/3 | 2/3 | 0.619794 | |
| c = 12.74900 ± 0.00026 | S2/Se | 4f | 1/3 | 2/3 | 0.619794 | |
| $R_p$ = 9.82 | | | | | | |
| $MoS_{0.6}Se_{1.4}$ | Mo1 | 2c | 1/3 | 2/3 | 0.25 | 1.41529 |
| a/b = 3.24862 ± 0.00003 | S1/S | 4f | 1/3 | 2/3 | 0.619845 | |
| c = 12.80990 ± 0.00012 | S2/Se | 4f | 1/3 | 2/3 | 0.619845 | |
| $R_p$ = 8.35 | | | | | | |
| $MoS_{0.4}Se_{1.6}$ | Mo1 | 2c | 1/3 | 2/3 | 0.25 | 1.42509 |
| a/b = 3.26225 ± 0.00007 | S1/S | 4f | 1/3 | 2/3 | 0.62126 | |
| c = 12.85850 ± 0.00045 | S2/Se | 4f | 1/3 | 2/3 | 0.62126 | |
| $R_p$ = 13.6 | | | | | | |
| $MoS_{0.2}Se_{1.8}$ | Mo1 | 2c | 1/3 | 2/3 | 0.25 | 0.844457 |
| a/b = 3.26941 ± 0.00003 | S1/S | 4f | 1/3 | 2/3 | 0.620912 | |
| c = 12.89550 ± 0.00017 | S2/Se | 4f | 1/3 | 2/3 | 0.620912 | |
| $R_p$ = 10.2 | | | | | | |
| $MoSe_2$ | Mo1 | 2c | 1/3 | 2/3 | 0.25 | 0.980848 |
| a/b = 3.28591 ± 0.00006 | S1/S | 4f | 1/3 | 2/3 | 0.621241 | |
| c = 12.93751 ± 0.00023 | S2/Se | 4f | 1/3 | 2/3 | 0.621241 | |
| $R_p$ = 11.6 | | | | | | |

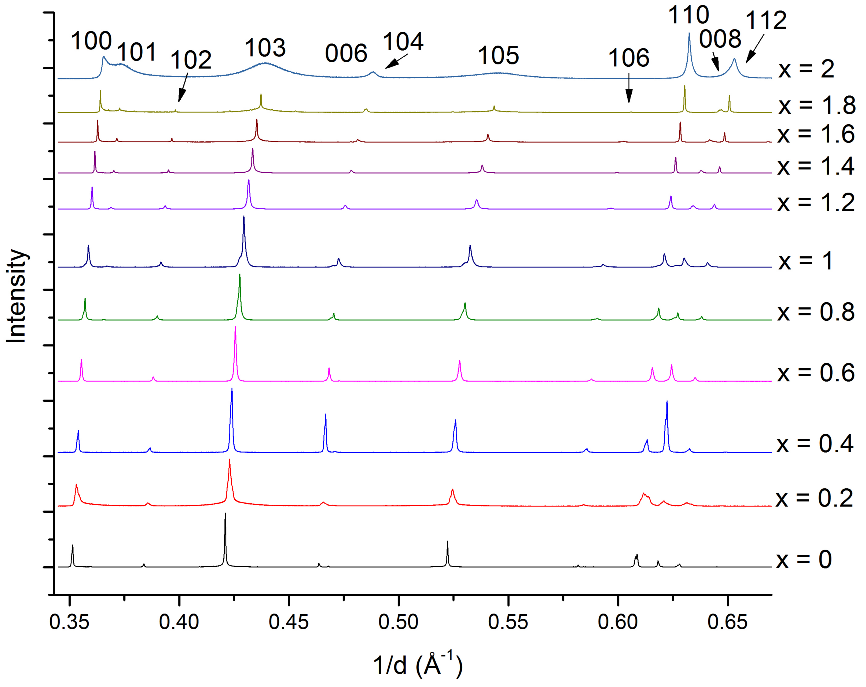


Figure S1.3. Synchrotron XRD patterns of $MoS_xSe_{2-x}$ solid solutions. The intensities are normalized to the 100 reflection of $MoSe_2$.

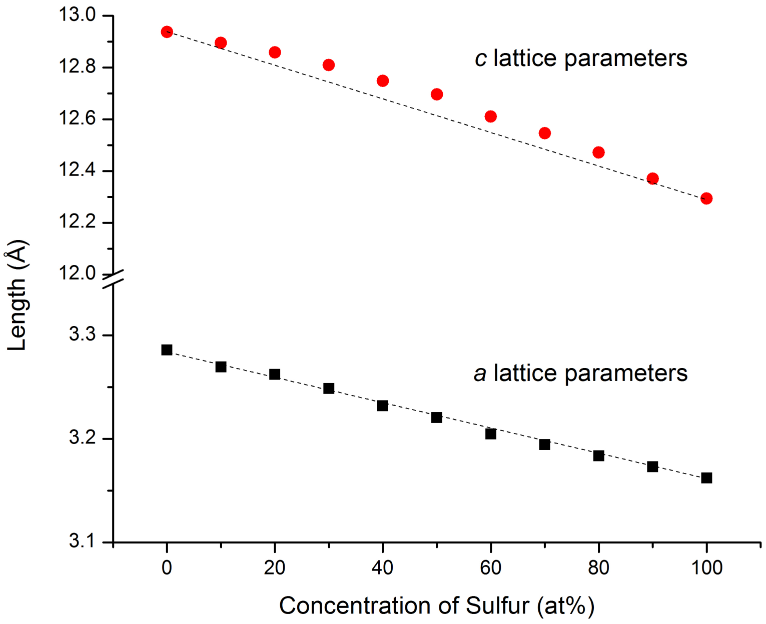


Figure S1.4. *a* and *c* lattice parameters of $MoS_xSe_{2-x}$ solid solutions as determined from Rietveld refinement of SXRD patterns. The black lines represent the theoretical values calculated from the end members by using Vegard's law. The error bars are smaller than the size of the plot symbols.

## 2. Characterization of crystal morphology and composition

All energy-dispersive X-ray spectroscopy spot and mapping experiments on the WSSe solid solutions samples were conducted using a calibrated FEI Quanta 200 Environmental Scanning Electron Microscope. The maps showed homogeneous distribution of W, S, and Se within the resolution of the technique for the bulk materials.

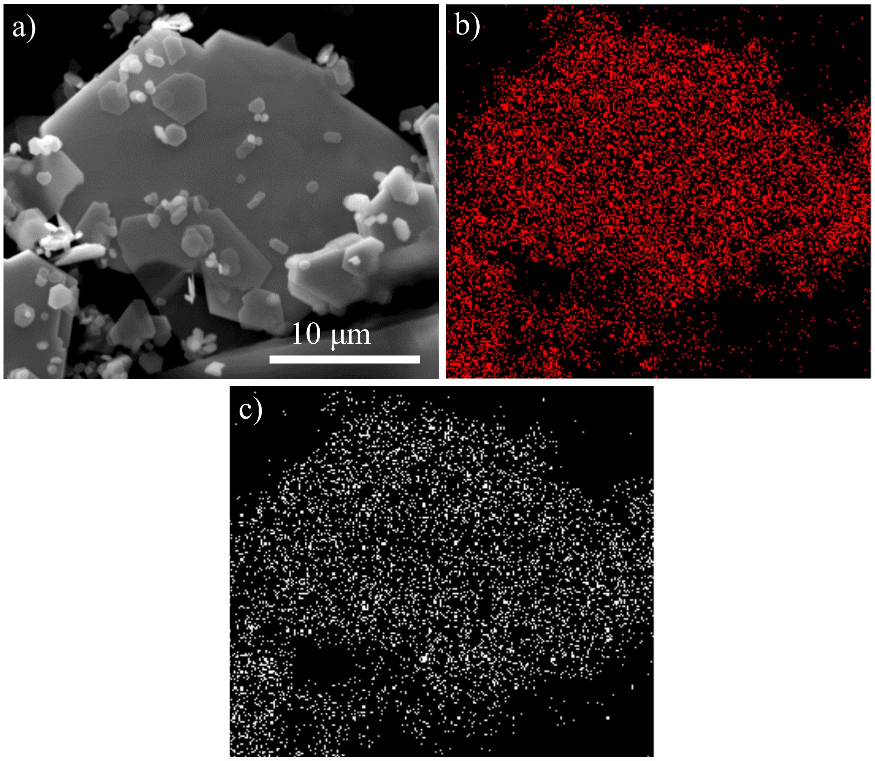


Figure S2. 1 SEM micrograph of $WS_2$ (a) and EDX mapping of sulfur (b) and tungsten [illegible]

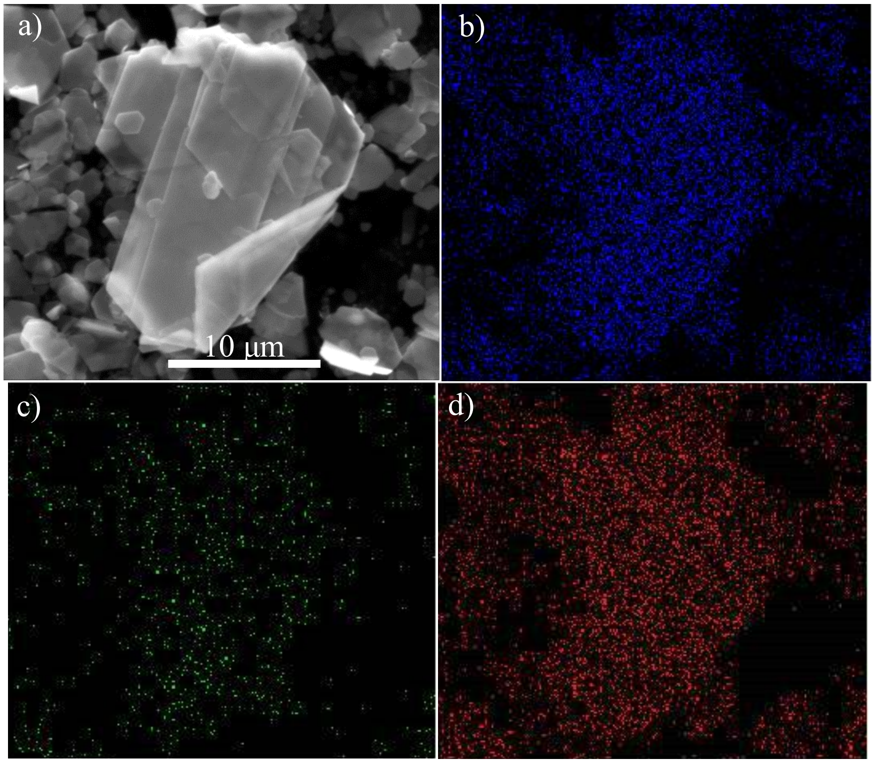


Figure S2.2 SEM micrograph of $WS_{1.8}Se_{0.2}$ (a) and EDX mapping of sulfur (b) , selenium (c) and tungsten (d)

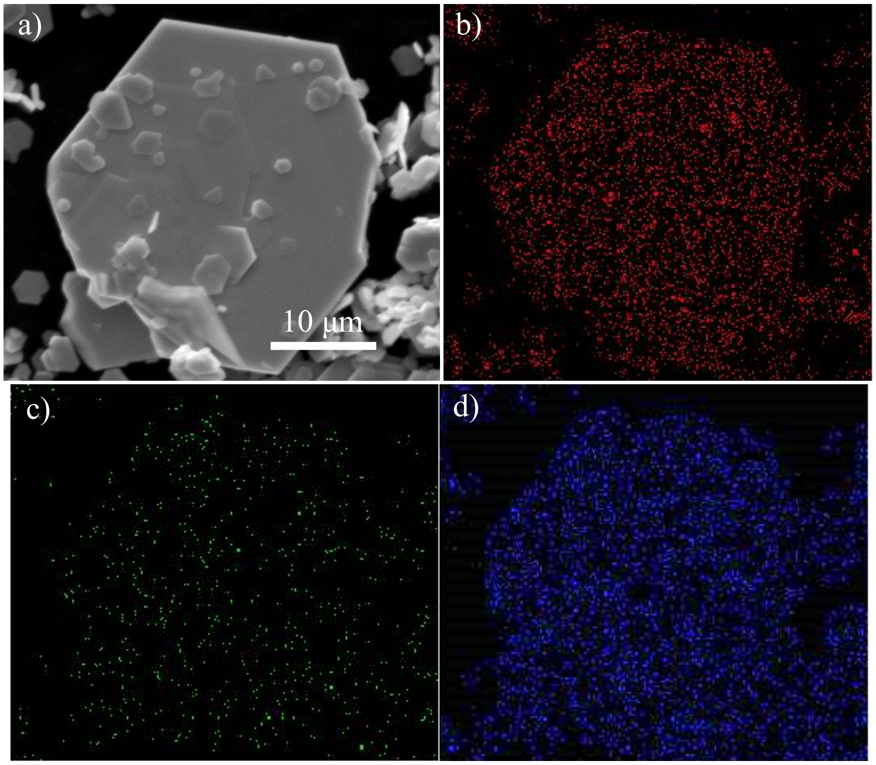


Figure S2.3 SEM micrograph of $WS_{1.6}Se_{0.4}$ (a) and EDX mapping of sulfur (b) , selenium (c) and tungsten (d)

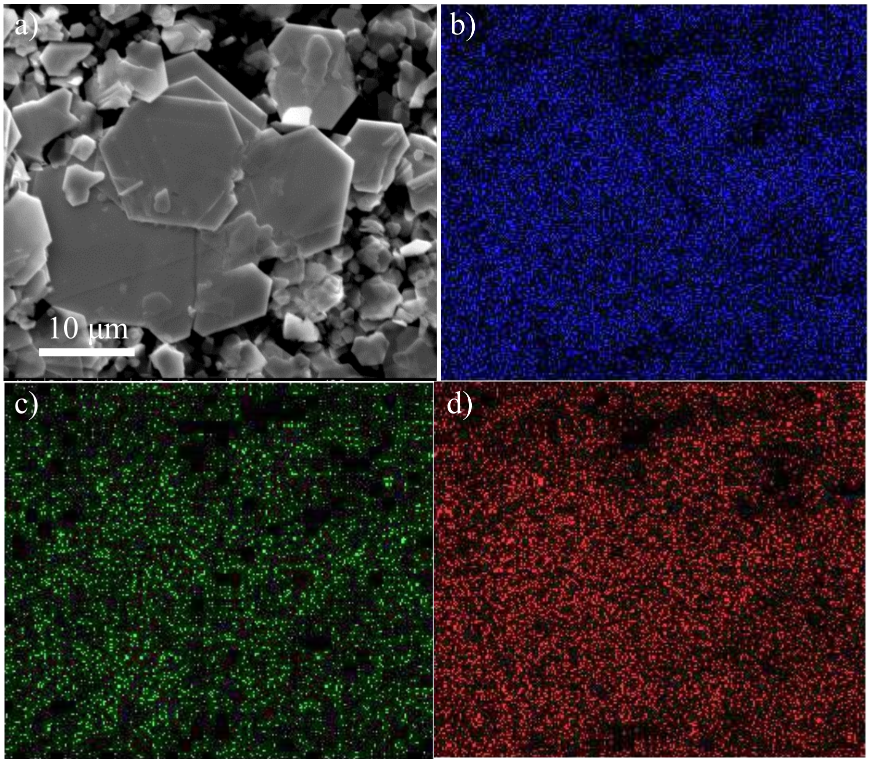


Figure S2.4 SEM micrograph of $WS_{1.2}Se_{0.8}$ (a) and EDX mapping of sulfur (b) , selenium (c) and tungsten (d)

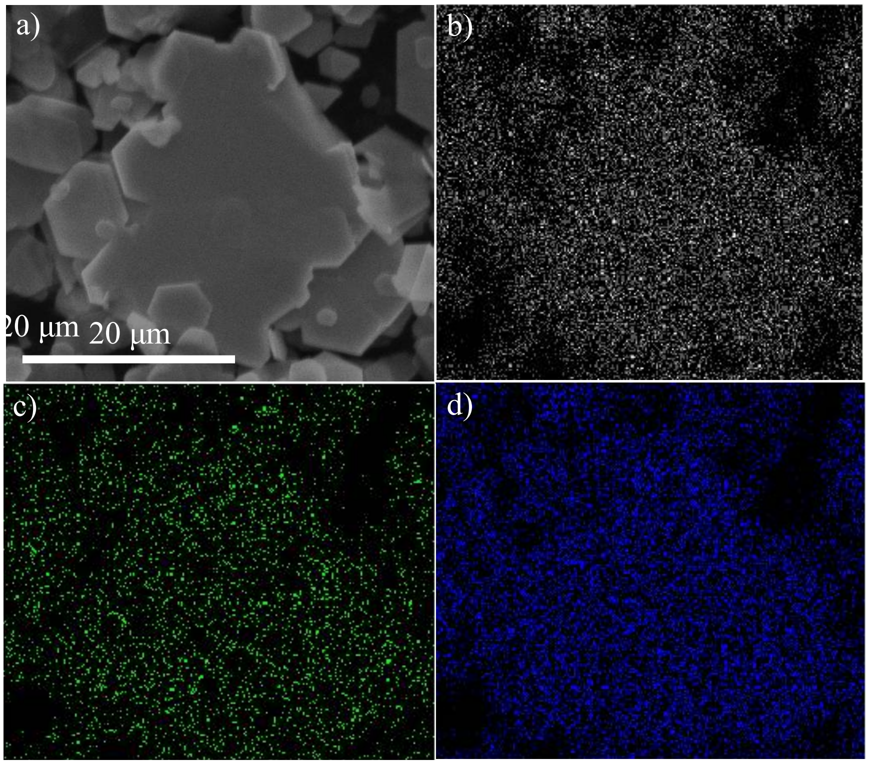


Figure S2.5 SEM micrograph of $WS_{1.4}Se_{0.6}$ (a) and EDX mapping of sulfur (b) , selenium (c) and [illegible]

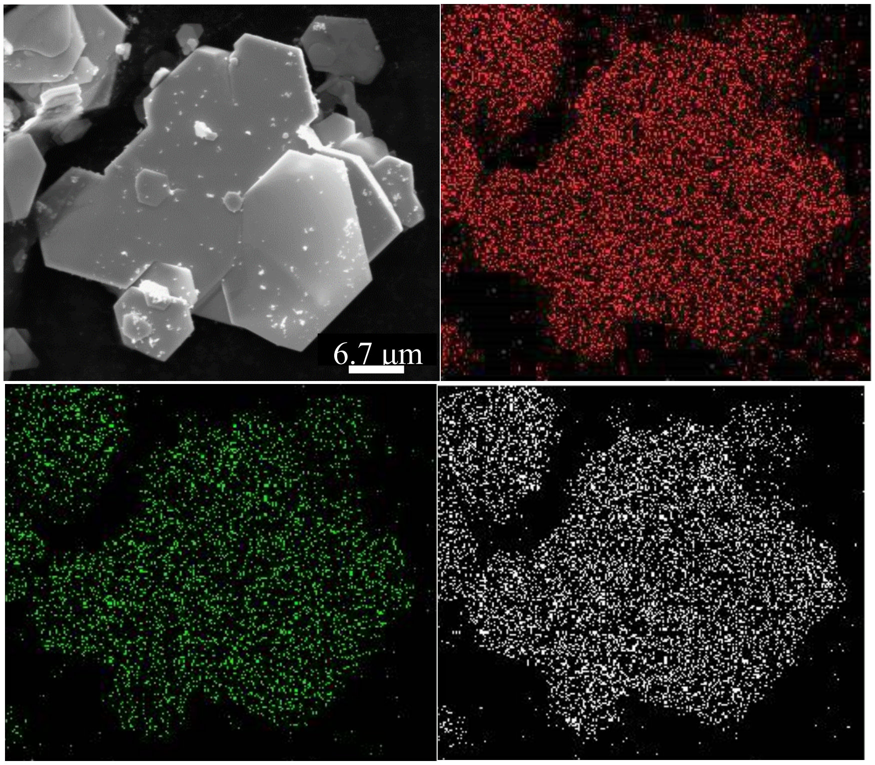


Figure S2.6 SEM micrograph of $WS_{0.8}Se_{1.2}$ (a) and EDX mapping of sulfur (b) , selenium (c) and tungsten (d)

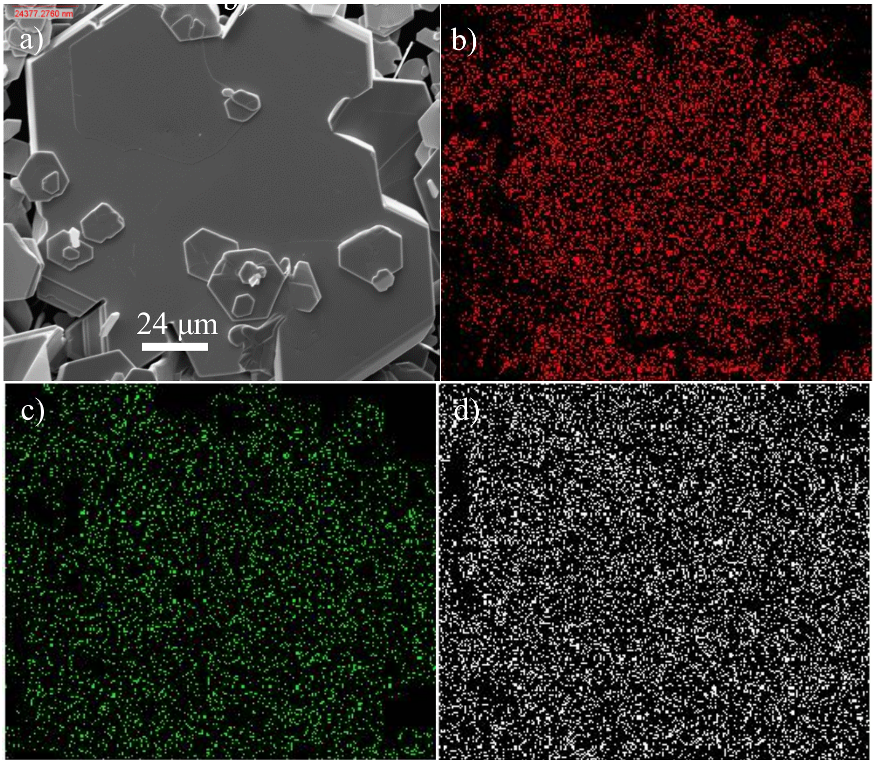


Figure S2.7 SEM micrograph of WSSe (a) and EDX mapping of sulfur (b) , selenium (c) and tungsten (d)

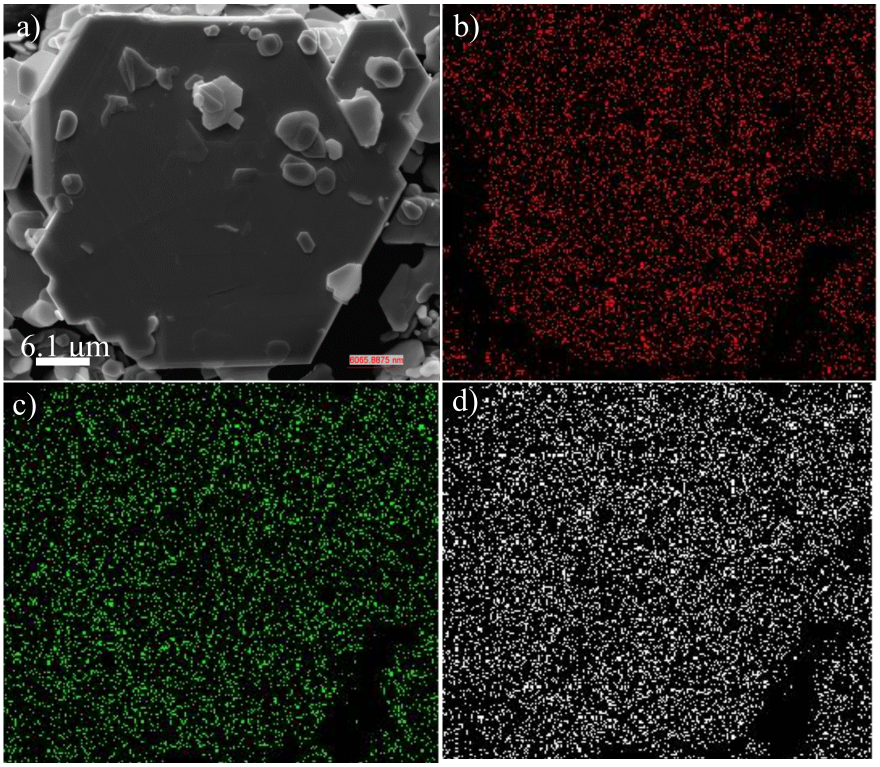


Figure S2.8 SEM micrograph of $WS_{0.4}Se_{1.6}$ (a) and EDX mapping of sulfur (b) , selenium (c) and tungsten (d)

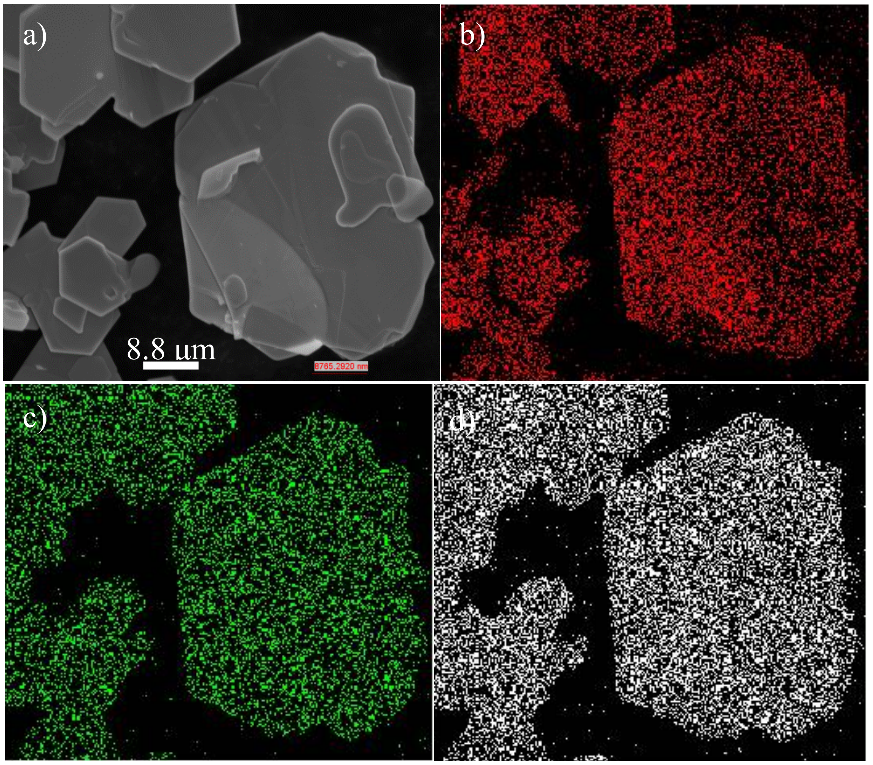


Figure S2.9 SEM micrograph of $WS_{0.6}Se_{1.4}$ (a) and EDX mapping of sulfur (b) , selenium (c) and tungsten (d)

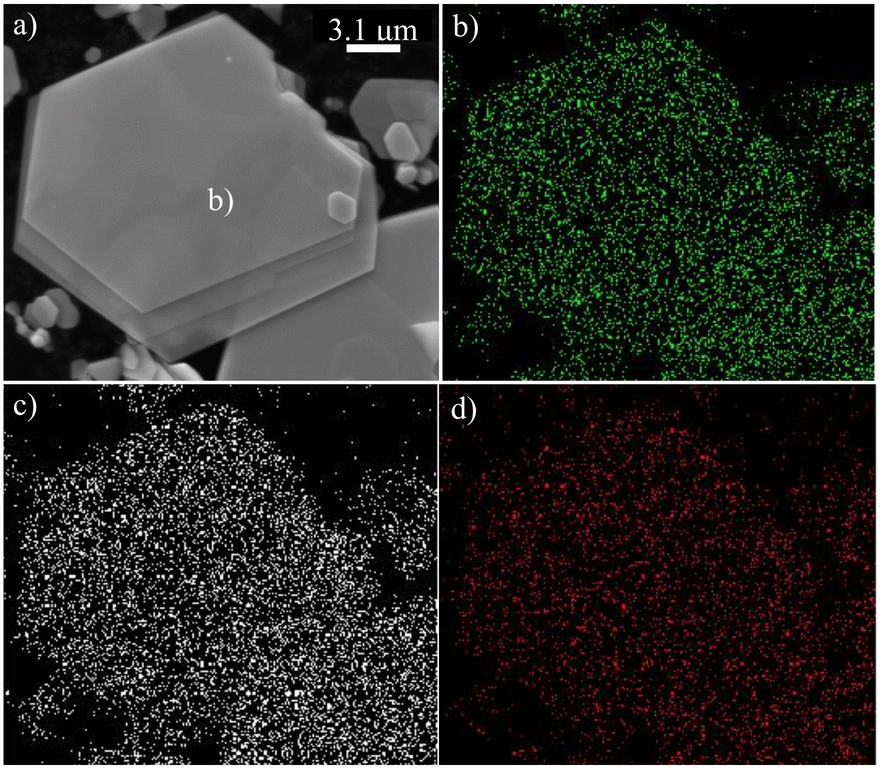

Figure S2.10 SEM micrograph of $WS_{0.2}Se_{1.8}$ (a) and EDX mapping of sulfur (b) , selenium (c) and tungsten (d)

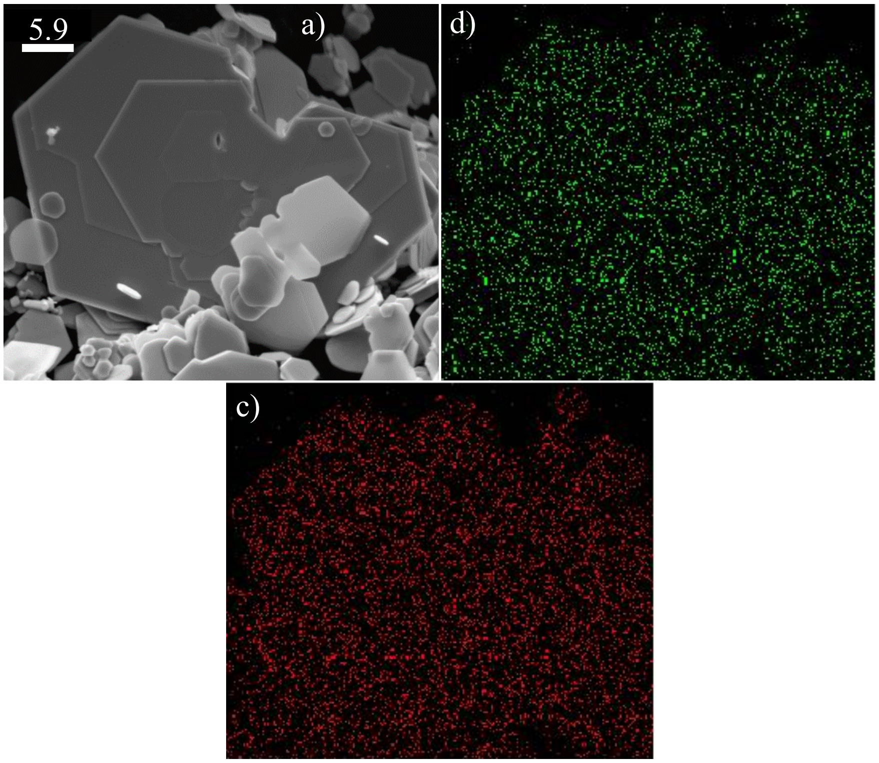

Figure S2.11 SEM micrograph of $WSe_2$ (a) and EDX mapping of sulfur (b) , selenium (c) and tungsten (d)

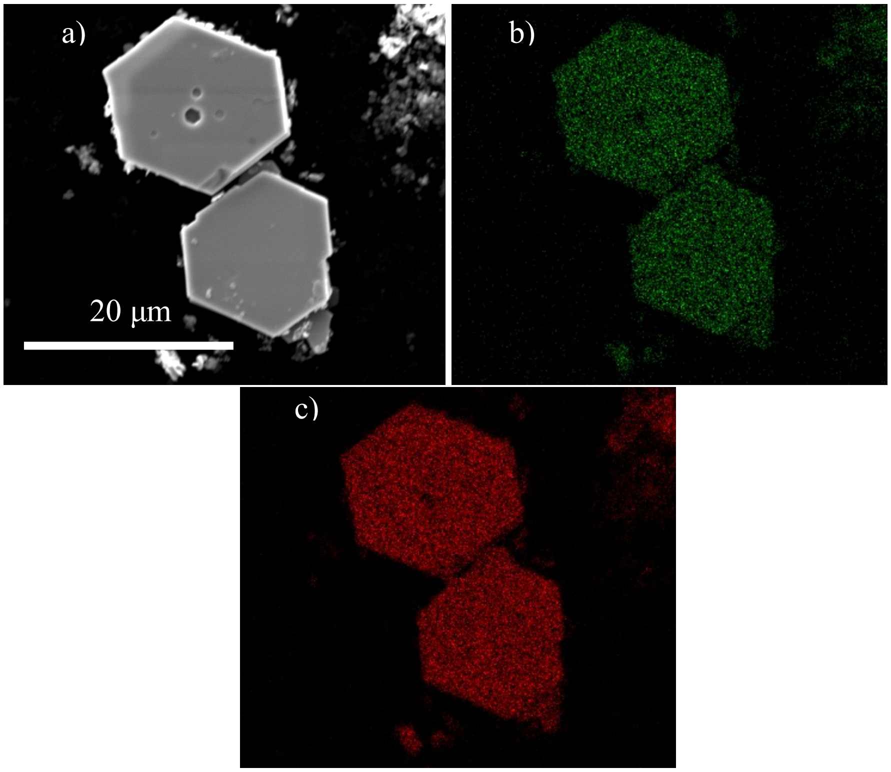


Figure S2.12. SEM micrograph of $MoSe_2$ (a) and EDX mapping of molybdenum (b) and selenium (c)

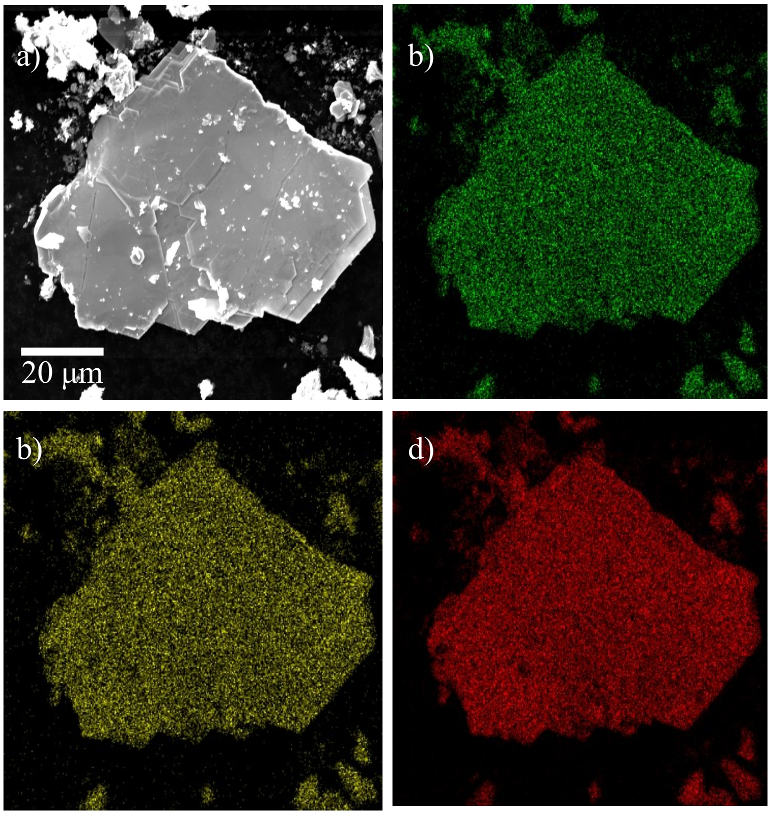

Figure S2.13 SEM micrograph of $MoS_{0.2}Se_{1.8}$ (a) and EDX mapping of molybdenum (b), sulfur (c) and selenium (d)

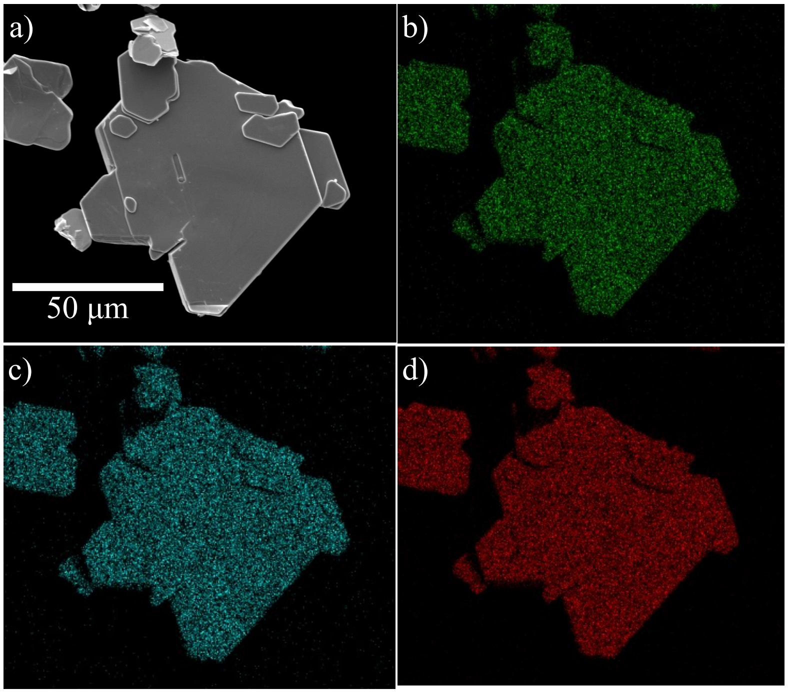

Figure S2.14. SEM micrograph of $MoS_{0.4}Se_{1.6}$ (a) and EDX mapping of molybdenum (b), sulfur (c) and selenium (d)

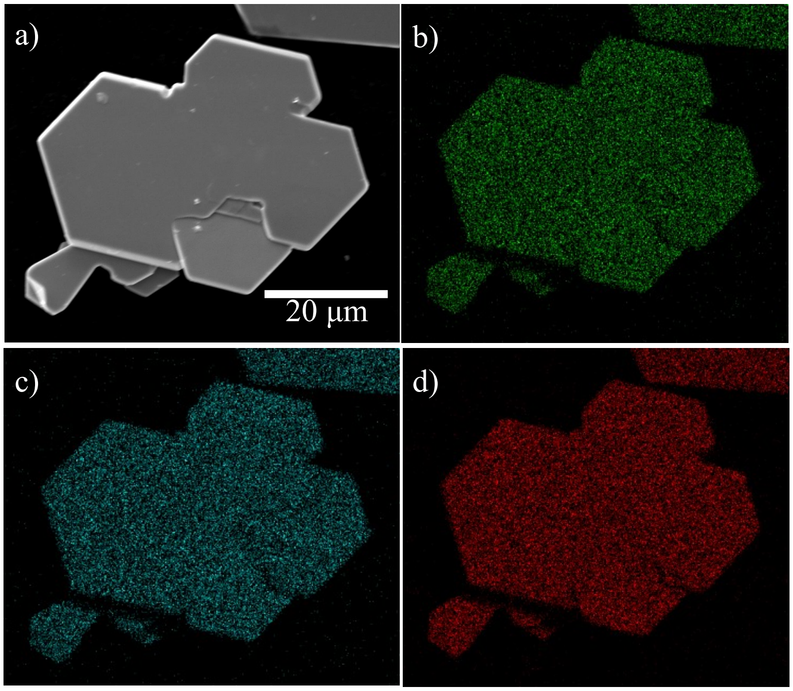


Figure S2.15. SEM micrograph of $MoS_{0.6}Se_{1.4}$ (a) and EDX mapping of molybdenum (b),

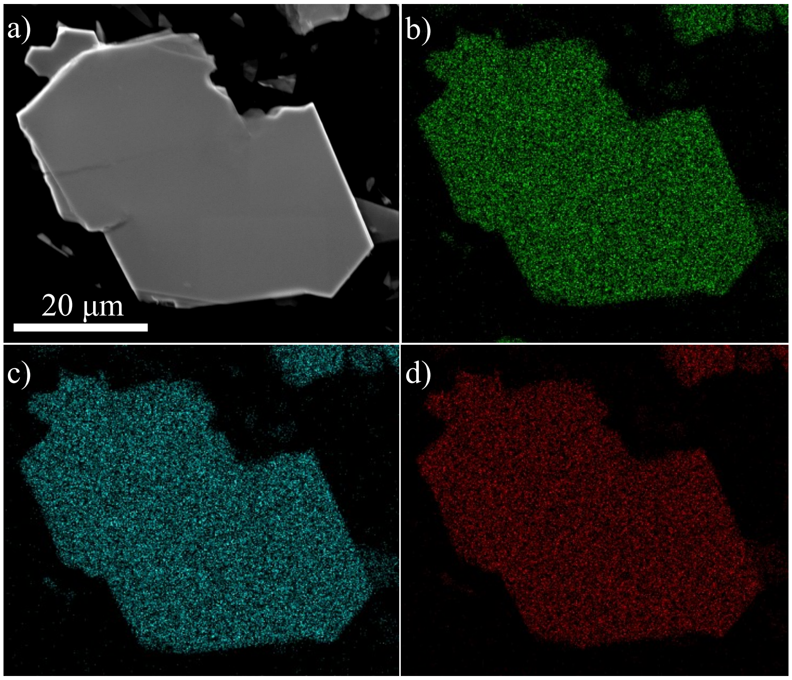


Figure S2.16 SEM micrograph of MoSSe (a) and EDX mapping of molybdenum (b), sulfur (c) and selenium (d)

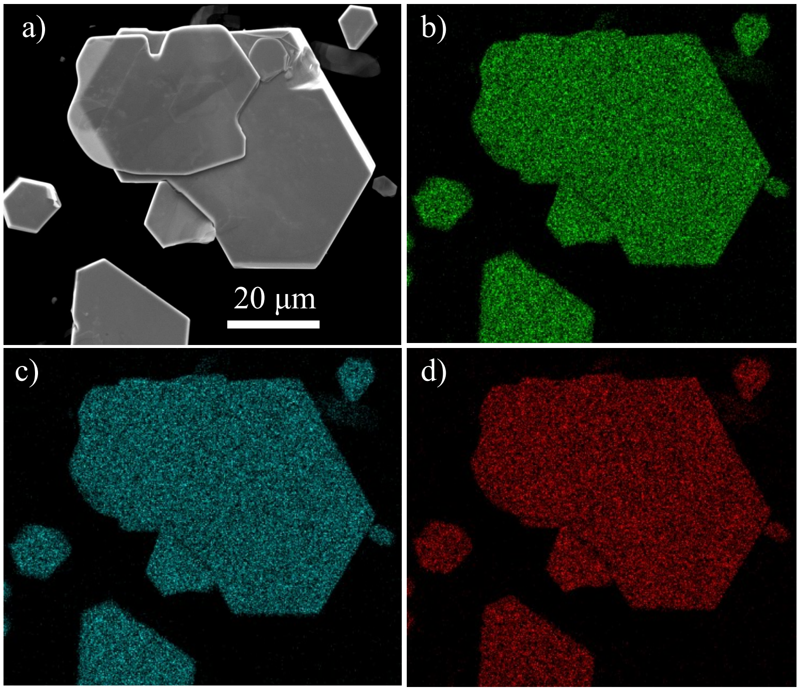

Figure S2.17. SEM micrograph of $MoS_{1.2}Se_{0.8}$ (a) and EDX mapping of molybdenum (b), sulfur (c) and selenium (d)

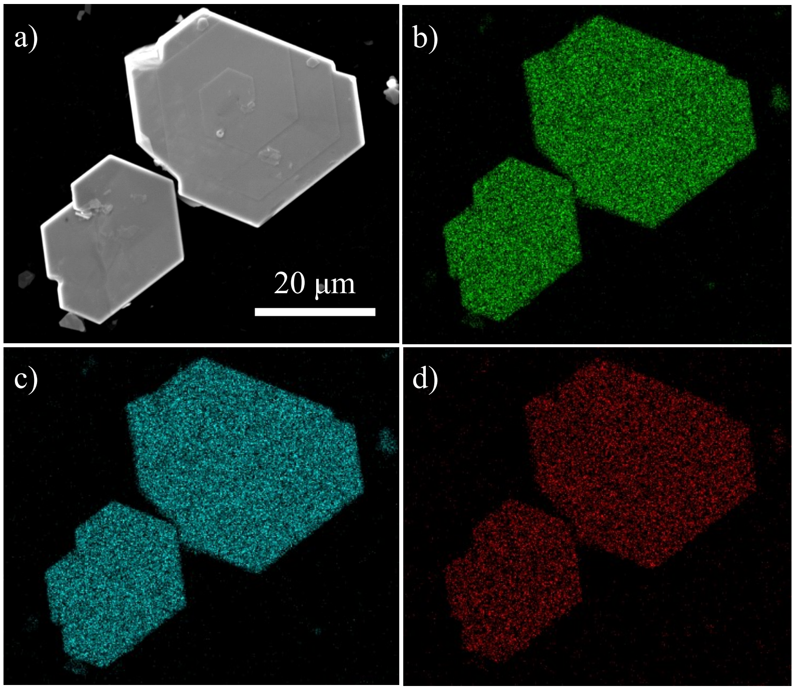


Figure S2.18. SEM micrograph of $MoS_{1.6}Se_{0.4}$ (a) and EDX mapping of molybdenum (b), sulfur (c) and selenium (d)

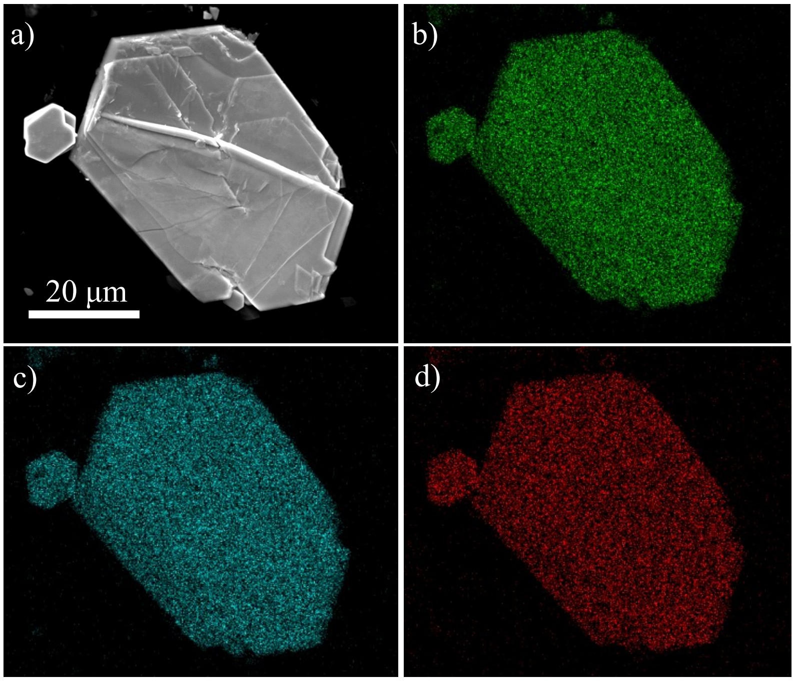


Figure S2.19 SEM micrograph of $MoS_{1.4}Se_{0.6}$ (a) and EDX mapping of molybdenum (b), sulfur (c) and selenium (d)

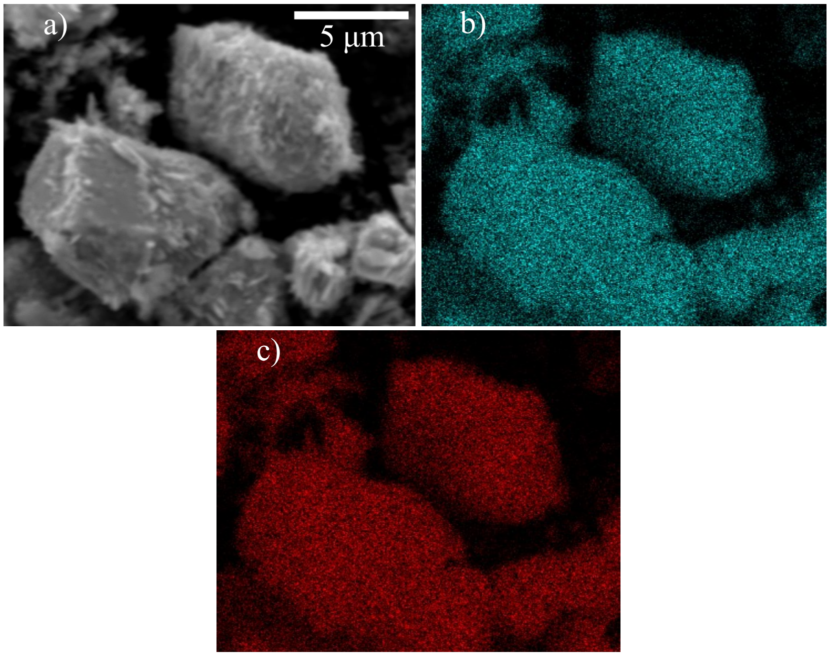


Figure S2.20. SEM micrograph of $MoS_2$ (a) and EDX mapping of molybdenum (b), sulfur (c)

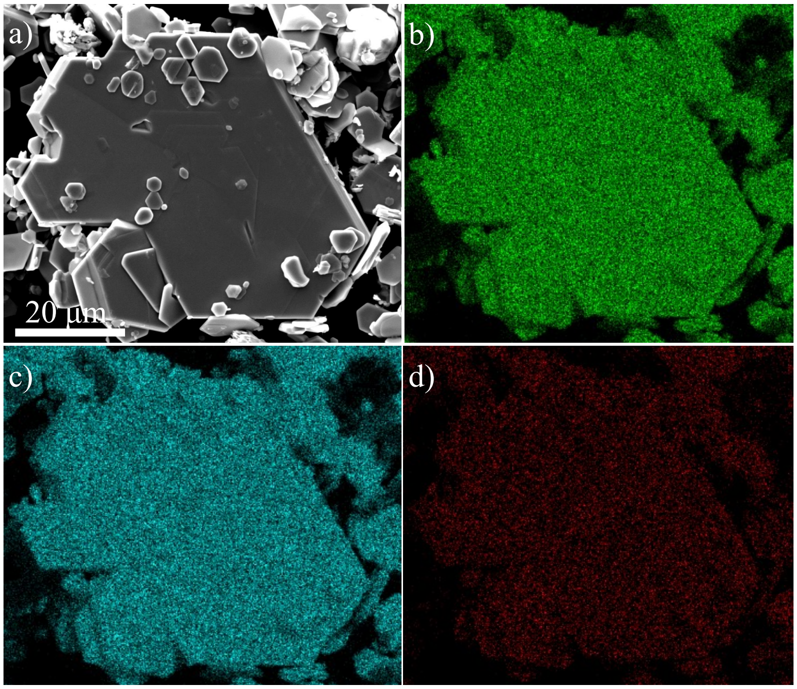


Figure S2.21. SEM micrograph of $MoS_{1.8}Se_{0.2}$ (a) and EDX mapping of molybdenum (b), sulfur (c) and selenium (d)

For each compound, EDX measurements were performed on five different particles. The average atomic percentages and standard deviations are reported in the tables below.

| Compound | Concentration (at%) | | |
|---|---|---|---|
| | Molybdenum | Sulfur | Selenium |
| $MoS_2$ | 31.0 ± 0.5 | 69.00 ± 0.5 | 0 |
| $MoS_{1.8}Se_{0.2}$ | 31.8 ± 0.8 | 61.0 ± 1.0 | 7.2 ± 0.9 |
| $MoS_{1.6}Se_{0.4}$ | 32.7 ± 2.1 | 54.6 ± 2.1 | 12.7 ± 2.3 |
| $MoS_{1.4}Se_{0.6}$ | 33.5 ± 1.7 | 45.6 ± 2.5 | 20.9 ± 2.6 |
| $MoS_{1.2}Se_{0.8}$ | 31.0 ± 2.8 | 39.3 ± 3.4 | 29.7 ± 1.3 |
| $MoS_{1.0}Se_{1.0}$ | 30.9 ± 3.6 | 35.1 ± 3.1 | 34.0 ± 1.8 |
| $MoS_{0.8}Se_{1.2}$ | 34.4 ± 3.1 | 26.2 ± 1.8 | 39.3 ± 3.1 |
| $MoS_{0.6}Se_{1.4}$ | 32.5 ± 2.1 | 21.9 ± 2.3 | 45.6 ± 1.7 |
| $MoS_{0.4}Se_{1.6}$ | 31.0 ± 3.8 | 10.5 ± 1.3 | 58.6 ± 4.3 |
| $MoS_{0.2}Se_{1.8}$ | 33.2 ± 4.6 | 7.8 ± 1.5 | 59.0 ± 3.3 |
| $MoSe_2$ | 30.5 ± 0.8 | 0 | 69.5 ± 0.8 |

Composition of $MoS_xSe_{2-x}$ solid solutions measured by SEM-EDX.

| Compound | Concentration (at%) | | |
|---|---|---|---|
| | Tungsten | Sulfur | Selenium |
| $WS_2$ | 33.7 ± 0.8 | 66.3 ± 0.8 | 0 |
| $WS_{1.8}Se_{0.2}$ | 32.8 ± 0.6 | 60.4 ± 1.1 | 6.8 ± 0.9 |
| $WS_{1.6}Se_{0.4}$ | 33.5 ± 0.6 | 53.2 ± 1.0 | 13.4 ± 0.6 |
| $WS_{1.4}Se_{0.6}$ | 33.8 ± 0.4 | 46.5 ± 0.6 | 19.7 ± 0.5 |
| $WS_{1.2}Se_{0.8}$ | 33.7 ± 0.7 | 40.1 ± 1.6 | 26.2 ± 2.0 |
| $WS_{1.0}Se_{1.0}$ | 33.0 ± 0.9 | 32.3 ± 1.9 | 34.7 ± 2.5 |
| $WS_{0.8}Se_{1.2}$ | 34.3 ± 0.2 | 26.7 ± 0.5 | 39.0 ± 0.6 |
| $WS_{0.6}Se_{1.4}$ | 33.3 ± 0.7 | 19.1 ± 0.7 | 47.6 ± 1.1 |
| $WS_{0.4}Se_{1.6}$ | 33.2 ± 1.3 | 12.9 ± 1.0 | 53.9 ± 1.9 |
| $WS_{0.2}Se_{1.8}$ | 34.6 ± 0.2 | 7.3 ± 0.1 | 58.1 ± 0.2 |
| $WSe_2$ | 33.3 ± 1.2 | 0 | 66.7 ± 1.2 |

Composition of $WS_xSe_{2-x}$ solid solutions measured by SEM-EDX.

### 3. HAADF-EDX mapping of atomic distributions of W, Mo, S, and Se

Monolayer regions were located under the TEM using the high angle annular dark field scanning transmission electron microscope. Elemental EDX mapping of W, Mo, S, and Se was performed on monolayers of $WS_{1.8}Se_{0.2}$. Examples are shown in Figures S3.1 and S3.2.

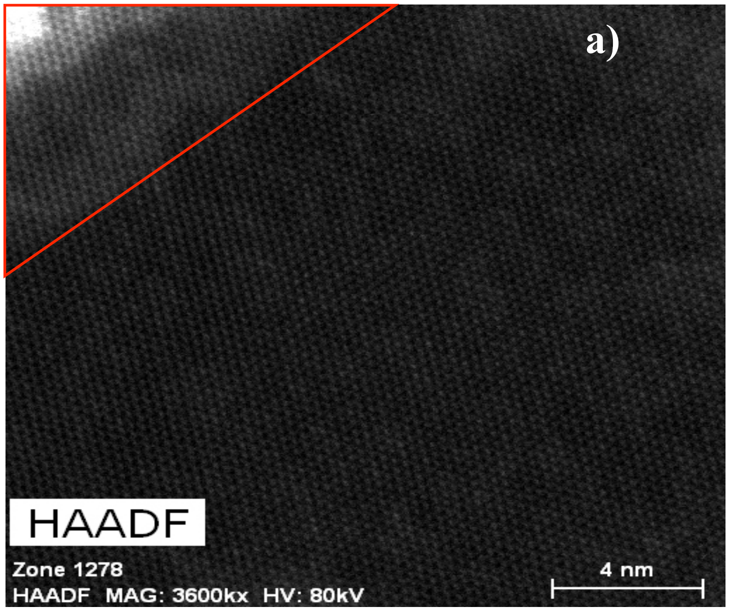


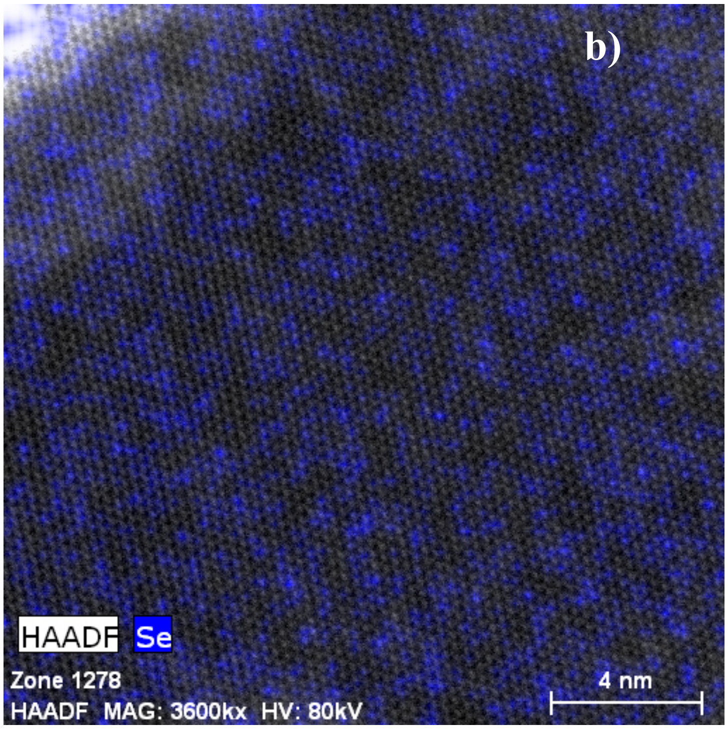

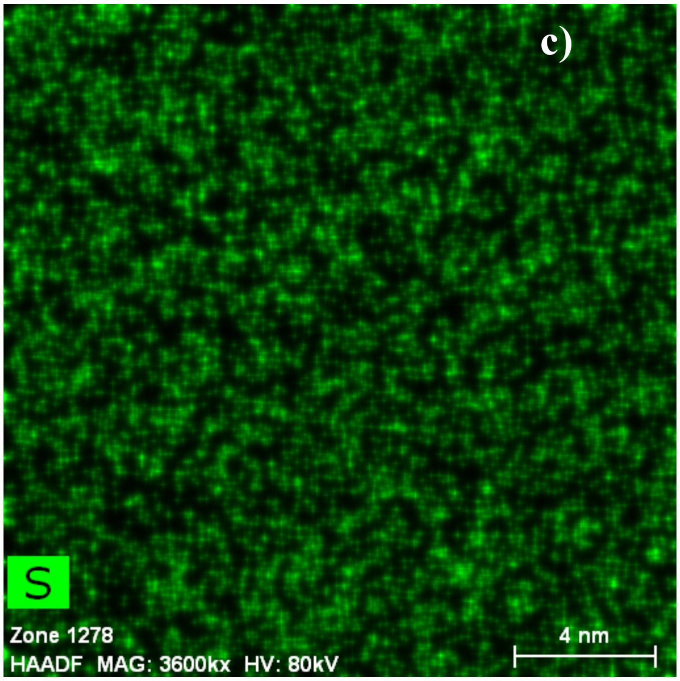


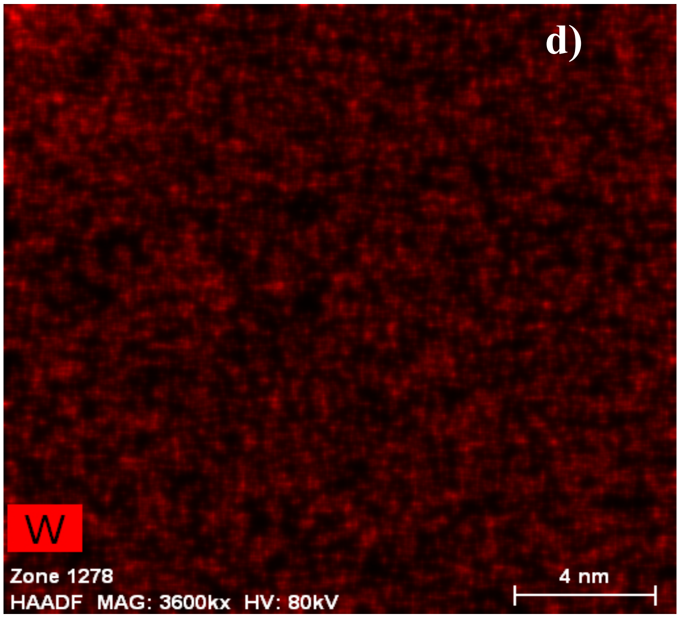


Figure S3.1. a) HAADF-STEM micrograph of $WS_{1.8}Se_{0.2}$. The corner outlined in red is a multilayer region. The rest of the image is a monolayer region. Elemental EDX maps of b) selenium overlayed on the HAADF-STEM micrograph of $WS_{1.8}Se_{0.2}$, c) sulfur d) tungsten.

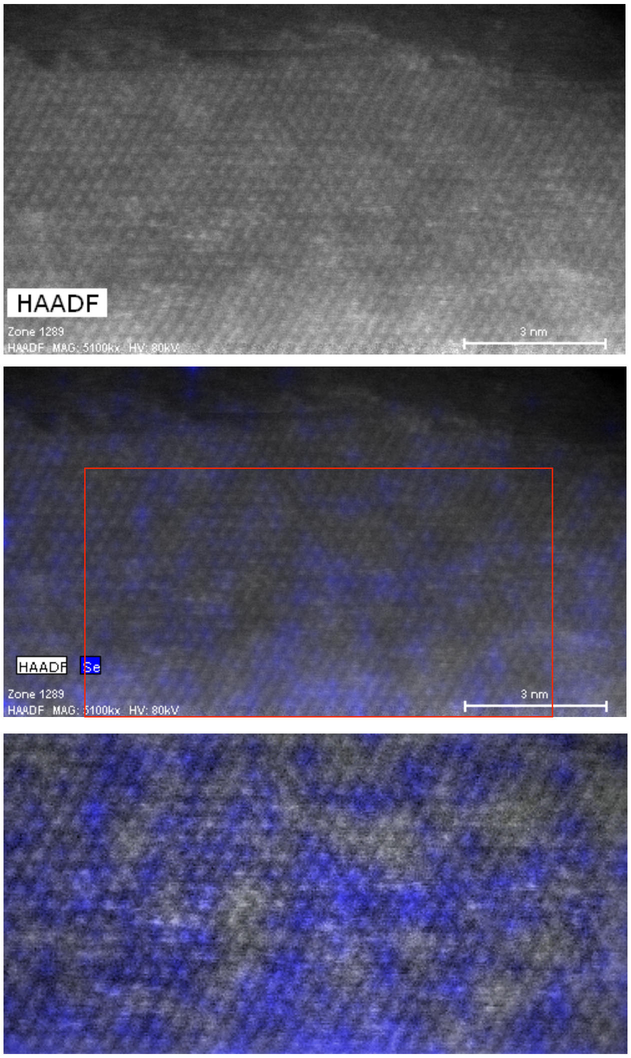


Figure S3.2 a) HAADF-STEM micrograph of $MoS_{1.8}Se_{0.2}$. Elemental EDX maps of b) selenium overlayed on the HAADF-STEM micrograph of $MoS_{1.8}Se_{0.2}$. The red box region in (b) was used for quantifying the degree of clustering within the monolayer. c) The enlarged red box region shown with contrast and brightness adjusted.

## 4. Calculation of the clustering parameter J from HAADF-EDX data

J was calculated from nearest-neighbor counting data in Fig. 9c according to

$$J = \frac{P_{observed}}{P_{random}} \times 100\%$$

where $P_{observed}$ and $P_{random}$ were obtained from:

$$P_{observed} = \frac{\sum_{i=0}^{6} (i \times N_{gray-i(blue)})}{N_{gray}}$$

and

$$P_{random} = \frac{N_{blue}}{N_{gray} - N_{blue}}$$

Here $N_{gray}$, $N_{blue}$, and $N_{gray\text{-}i(blue)}$ are the number of gray-colored metal atoms (coordinated only to sulfur), the number of blue-colored metal atoms (coordinated to one or more Se), and the number of gray-colored W atoms surrounded by i (i = 0 to 6) blue-colored metal atoms, respectively.
